# Large-scale simulation of traffic flow using Markov model

**DOI:** 10.1371/journal.pone.0246062

**Published:** 2021-02-09

**Authors:** Renátó Besenczi, Norbert Bátfai, Péter Jeszenszky, Roland Major, Fanny Monori, Márton Ispány

**Affiliations:** Department of Information Technology, University of Debrecen, Debrecen, Hungary; Southeast University, CHINA

## Abstract

Modeling and simulating movement of vehicles in established transportation infrastructures, especially in large urban road networks is an important task. It helps in understanding and handling traffic problems, optimizing traffic regulations and adapting the traffic management in real time for unexpected disaster events. A mathematically rigorous stochastic model that can be used for traffic analysis was proposed earlier by other researchers which is based on an interplay between graph and Markov chain theories. This model provides a transition probability matrix which describes the traffic’s dynamic with its unique stationary distribution of the vehicles on the road network. In this paper, a new parametrization is presented for this model by introducing the concept of two-dimensional stationary distribution which can handle the traffic’s dynamic together with the vehicles’ distribution. In addition, the weighted least squares estimation method is applied for estimating this new parameter matrix using trajectory data. In a case study, we apply our method on the Taxi Trajectory Prediction dataset and road network data from the OpenStreetMap project, both available publicly. To test our approach, we have implemented the proposed model in software. We have run simulations in medium and large scales and both the model and estimation procedure, based on artificial and real datasets, have been proved satisfactory and superior to the frequency based maximum likelihood method. In a real application, we have unfolded a stationary distribution on the map graph of Porto, based on the dataset. The approach described here combines techniques which, when used together to analyze traffic on large road networks, has not previously been reported.

## Introduction

In the past decade, research and development of smart city applications have become an active topic [1, 2]. These services contain solutions such as intelligent city planning, crowdsourcing, as well as crisis and disaster management [3]. These applications will also both generate and make use of big data which will arise from the wide availability of cloud computing and IoT applications [4]. By the year 2050, 68% of Earth’s population is expected to live in urban areas [5]. City infrastructures will face new challenges from many factors; one such factor is urban traffic. Moreover, a solution for the problem of air pollution and congestion is highly demanding [6–8]. In the recent years, the research and development of intelligent transportation systems (ITS) in the context of smart cities have become a vivid topic [9, 10]. In the near future, a smart city ITS application may also have a requirement to support the operation of both self-driving and electric cars [11–13].

This research follows and contributes to our development of a traffic simulation platform initiative called rObOCar World Championship (or OOCWC for short) [13–17]. The OOCWC is a multiagent-oriented environment for creating urban traffic simulations and for investigating the relationship between smart cities and self-driving cars. The traffic simulations are performed by one of its components called *Robocar City Emulator* (RCE). We extract geographical information from OpenStreetMap (OSM) and transform this data into a routing map graph. The simulation takes place on a rectangular part of the OSM. The traffic simulation model of the RCE is based on the Nagel-Schreckenberg cellular automata model [18]. We slice all graph edges for parts 3 meters long, so the length of each cell is *l* = 3*m*. Each edge has only one lane and up to one car can occupy a cell at every time step Δ*t* = 0.2*s*; therefore, each simulation unit moves with fixed speed *v* = 54*km*/*h*. During the simulation, we can observe how the distribution of the cars changes. In the original implementation, the simulation algorithm moves the cars by random walk. So, when a car arrives at a graph vertex (i.e. intersection), it selects the next edge (i.e. next road segment) according to uniform distribution and is delivered to the next edge if the first cell of the next edge is free. This model is somewhat similar to that is used in [7]. One statistic that can represent this distribution to test the stationarity is the order of the streets based on the number of cars on them. An important aspect is that the order of the street should remain the same during the simulation when it is already in a steady state. In paper [14], we showed that in the original edition of the OOCWC the order of the streets changes almost randomly even when the simulation has been running for a long time, so the requirement of stationarity does not hold. In this paper, a method is proposed to answer this problem. For a detailed description of the operation of RCE, see paper [13]. There exist several traffic simulation platforms (Multi-Agent Transport Simulation [19], Simulation of Urban Mobility [20], Aimsun (see https://www.aimsun.com/), PTV Vissim (see http://vision-traffic.ptvgroup.com/en-us/products/ptv-vissim/)). Although these applications are widely used in traffic analysis and planning, the main focus of their simulation algorithms is on microscopic traffic events. In contrast, our software system focuses only on the traffic flow of the whole city, or, to be more precise, the traffic graph.

A fundamental requirement in developing a traffic simulation algorithm which controls the simulated cars is to hold the real distribution of cars in a stationary way, see our previous paper [14]. Another aspect is how we are able to fit this algorithm by estimating its parameters based on real data, which has the form of trajectories. Our result presented in this paper is an answer to these problems. In [21], see also [22] and [23], a stochastic model is proposed which can handle the traffic in an urban network by using a mathematically rigorous method. This model is based on discrete time Markov chain on the road graph which plays the role of the state space. In the traffic interpretation, the transition probability matrix describes the dynamic of the traffic while its unique stationary distribution corresponds to the traffic equilibrium or steady state on the road network. In this steady-state, the distribution of vehicles remains invariant locally in time under the transport dynamic. Thus, this stationary distribution of the Markov chain can be interpreted as the momentary “true” distribution of the vehicles on the road network.

Note that the joint application of Markov chains and large graphs to analyze the behavior of complex systems is well known in several fields, e.g., distributed systems [24], geophysics [25] and biology [26]. Several approaches exist for short-term traffic flow prediction. These models are based on many techniques including Box-Jenkins time-series analyses with ARIMA model [27–30], Kalman filter theory [31, 32], non-parametric methods (k-NN, kernel, local regression) [33–36], exponential smoothing [37, 38], spectral analysis [39] or wavelets [40–42]. In addition, several approaches use machine learning and data mining techniques, such as support vector regression [43], artificial neural networks [44–46], Bayesian networks [47] or deep learning [48]. Some applications can be found based on computational intelligence techniques, e.g., linear genetic programming [49] or fuzzy logic [50–52], but seldom can we find approaches based on Markov models, see [53] and [21] mentioned previously.

Our contributions in this paper are as follows. Based on [21], we introduce the concepts of a Markov random walk, which describes the motion of an individual vehicle, and Markov traffic, which describes the entire traffic on the road network, respectively. We derive the stationary distribution of the Markov traffic as a multinomial distribution, see formula (3). We present how the ergodic theory of finite Markov chains can be applied to prove the ergodicity of Markov traffic model which implies that complex traffic events can be approximated well by the help of the stationary distribution of a Markov chain on the road network. This result also yields the theoretical ground of our simulation algorithm. We reparametrize the model by introducing the concept of two-dimensional stationary distribution which possesses equi-distributed marginals that are the unique stationary distribution of the transition probability matrix, respectively. To estimate this parameter matrix the weighted least squares (WLS) estimation as a kind of composite (quasi-) likelihood methods is applied, see [54]. In Theorem 2, we show that the WLS estimator of the two-dimensional stationary distribution can be expressed explicitly. Moreover, this estimation method provides a computationally effective technique on a large scale since the MapReduce paradigm can be easily applied to it. Finally, we present how a city-controlled IT solution can be developed which is able to simulate the traffic on a road network that fits to real world data.

## Modeling traffic flow by Markov chains on graphs

In this section, we overview a traffic simulation model that uses tools from graph theory and Markov chains. First, we outline the basic concepts in the fields of graph theory and finite Markov chains. Then, we describe the proposed model called “Markov traffic” shortly. Subsection after that is devoted to the ergodicity of Markov traffic model. As a case study, we use a publicly available trajectory dataset, namely, the Taxi Trajectory Prediction dataset (see https://www.kaggle.com/c/pkdd-15-predict-taxi-service-trajectory-i). After, we outline the key points of how we selected and processed the trajectory data. Finally, we describe how we process OSM data and build a traffic graph from this data.

### Road network: Basic concepts and notation

In this subsection, we outline the concepts of graph theory that are necessary for modeling traffic flow. A standard textbook on graph theory is [55].

Let *G* = (*V*, *E*) be a directed graph (digraph) where *V* and *E* denote the set of vertices or nodes and the set of directed edges or arcs or arrows of the graph, respectively. In the sequel, vertices are denoted by *u*, *v*, *w*, edges are denoted by *e*, *f*, *g*. For a directed edge *e* = (*v*, *w*) ∈ *E* we also use the notation *v* → *w*. We suppose that *G* is a simple digraph in the sense that it does not contain multiple arrows and loops. Multiple arrows means two or more edges that connect the same two vertices in the same direction. The edge (*v*, *v*), *v* ∈ *V*, is called a loop, i.e., it connects the vertex *v* to itself. The digraph *G*, called road network in this paper, represents the road system of a city. More precisely, we start from the following definition, see [56].

**Definition 1**. A **road network** *G* is a simple directed graph, *G* = (*V*, *E*), where *V* is a set of nodes representing the **terminal point**s of road segments, and *E* is a set of directed edges denoting road segments.

A **road segment** *e* = (*v*, *w*) ∈ *E* is a directed edge in the road network graphs, with two terminal points *v* and *w*. The vehicle flow on this edge is from *v* to *w*.

**Fig 1:**
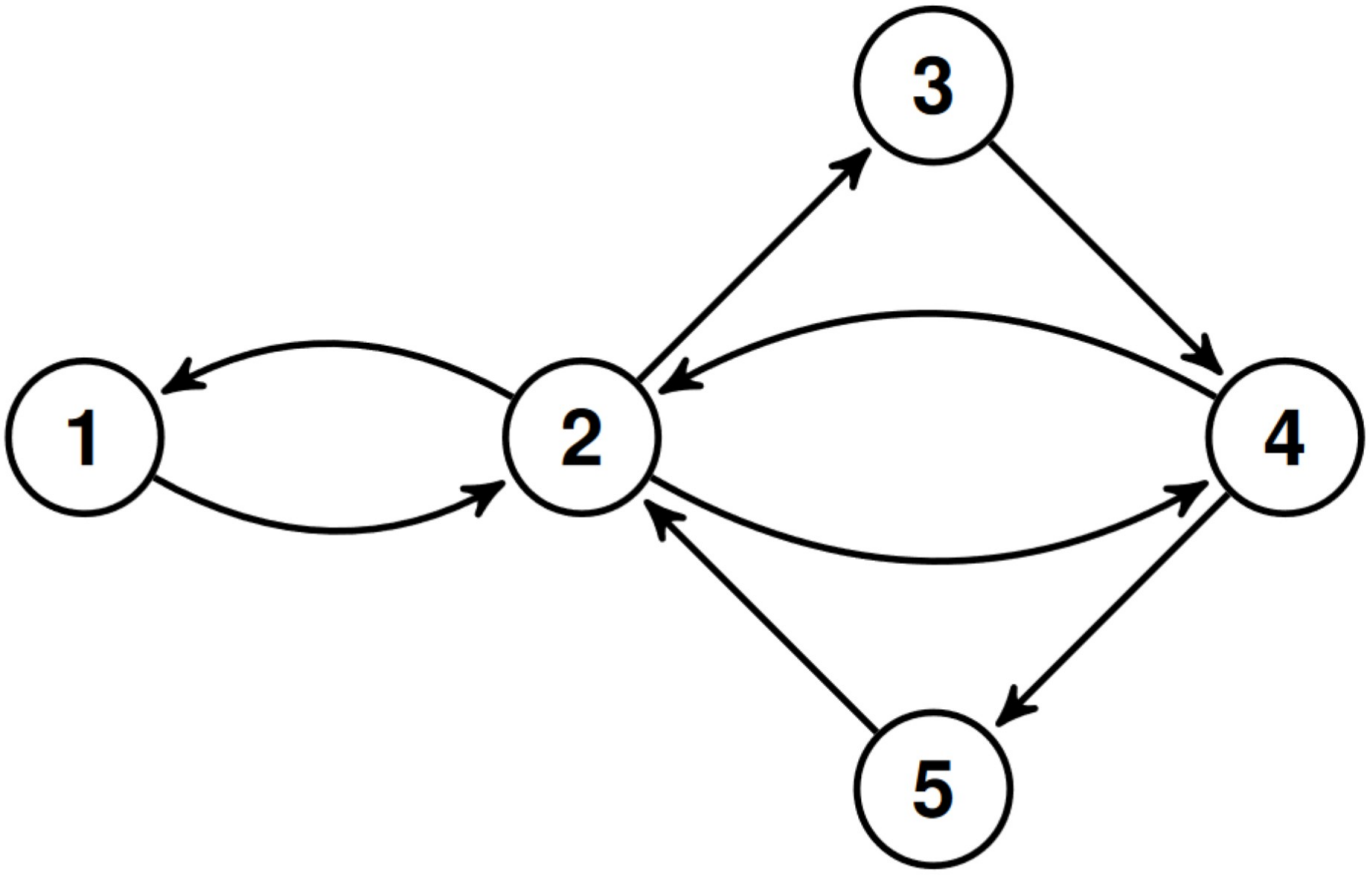
A simple road network.

Note that the simplicity of the graph model of an existing physical road network is clearly guaranteed if the resolution of the network is enough high. The resolution of a road network can be increased by introducing new terminal points on road segments splitting them into smaller road segments. By locking out the loops we can avoid that vehicles can move in an infinite cycle remaining persistently at the same node. Later however, when we define the traffic flow on a road network, we allow “virtual” loops to ensure that vehicles may remain at the same node or edge of the road network after a time step. Let *S* denote the set of loops in *G*, i.e., *S* ≔ {(*v*, *v*)|*v* ∈ *V*}. Since *G* is simple *E* ∩ *S* = ∅. Fig 1 presents a simple example for road network.

For a digraph *G* = (*V*, *E*) another digraph can be associated by the following way. Let the set *V*′ of vertices of this new digraph be the set of directed edges *E* of *G* and let the set *E*′ of its directed edges consist of the ordered pair (*e*, *f*) where *e*, *f* ∈ *E* such that there exist *u*, *v*, *w* ∈ *V* that *e* = (*u*, *v*) and *f* = (*v*, *w*), i.e., *u* → *v* → *w* is a path (dipath) in *G* of length 2. This associated digraph is called a directed line graph, see Section 4.5 in [55], shortly line digraph, or line road network (network line graph, see [57]), and it is denoted by L(*G*) = (*V*′, *E*′). The elements of *E*′ can be described by triplets (*u*, *v*, *w*), where *u*, *v*, *w* ∈ *V*, (*u*, *v*), (*v*, *w*) ∈ *E*, and for a directed edge in L(*G*) we may use the notation (*u*, *v*)→(*v*, *w*) too.

The basic difference between the digraph and line digraph views of a road network is that the former assigns the vehicles moving in a city to the vertices while the latter to the edges. One can refer the former as first-order (primal) network while the latter as second-order (dual) network, see [58, 59]. These two kinds of graphs are both useful because in a road network, certain measurements are associated with the crossings (vertices), and certain measurements are associated with the road segments (directed edges). When we are concerned with comparing measurements associated with crossings, then we will be concerned with the adjacency relationships of crossings, and so with the road network. However, when we are concerned with measurements associated with road segments we will be concerned with the adjacency relationships of road segments, and so our analyses will involve the line road network.

**Fig 2:**
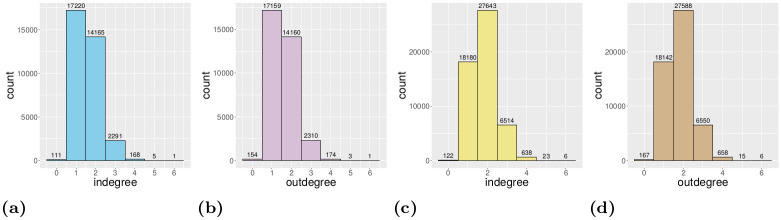
The degree distribution histograms of the Porto map traffic graph. a: Indegree distribution (vertices). b: Outdegree distribution (vertices). c: Indegree distribution (edges). d: Outdegree distribution (edges).

The degree distributions of the digraphs *G* and L(*G*), respectively, are given in the following way. For *v* ∈ *V* define *v*^−^ ≔ {*e* ∈ *E*|∃*u* ∈ *V*: *e* = (*u*, *v*)} and *v*^+^ ≔ {*e* ∈ *E*|∃*w* ∈ *V*: *e* = (*v*, *w*)}, i.e., *v*^−^ and *v*^+^ are the sets of arrows in and out the node *v*, respectively. Note that *deg*^−^(*v*) = |*v*^−^| and *deg*^+^(*v*) = |*v*^+^| is the indegree and outdegree of *v*, respectively, where |⋅| denotes the cardinality of a set. For all *i* = 0, 1, 2, … define $n_{i}^{+}≔\left|\left\{v\in V\;|\;deg^{+}\left(v\right)=i\right\}\right|$. Then, the pairs $(i,n_{i}^{+}),i=0,1,2,\ldots$, form the frequency histogram for the outdegree distribution of *G*. The indegree frequency histogram is defined similarly as $(i,n_{i}^{-}),i=0,1,2,\ldots$, where $n_{i}^{-}≔\left|\left\{v\in V\;|\;deg^{-}\left(v\right)=i\right\}\right|$. On the other hand, for all *i* = 0, 1, 2, …, define $m_{i}^{+}≔\sum_{v\in G_{i}^{+}}deg^{-}\left(v\right)$ where $G_{i}^{+}≔\left\{v\in V\;|\;deg^{+}\left(v\right)=i\right\}$. Then, the pairs $(i,m_{i}^{+}),i=0,1,2,\ldots$, form the frequency histogram for the outdegree distribution of L(*G*). Note that $n_{i}^{+}=\left|G_{i}^{+}\right|$ for all *i*. Similarly, the pairs $(i,m_{i}^{-}),i=0,1,2,\ldots$, form the frequency histogram for the indegree distribution of L(*G*) where $m_{i}^{-}≔\sum_{v\in G_{i}^{-}}deg^{+}\left(v\right)$ and $G_{i}^{-}≔\left\{v\in V\;|\;deg^{-}\left(v\right)=i\right\}$. (Note that $n_{i}^{-}=\left|G_{i}^{-}\right|$ for all *i*.) One can easily see that the supports of the two indegree (outdegree) histograms are the same. For the Porto example (described later in this paper), the above mentioned degree distributions can be seen in Fig 2. These histograms corroborate the fact that the Porto’s road network, as all city’s road network, is a sparse graph since there is no node with higher in- and outdegree than 6 and the ratio of the number of edges and the number of nodes is less than 2, see Fig 5.

Finally, we recall some topological properties of digraph *G*. For a pair *u*, *v* ∈ *V*, *u* ≠ *v*, *v* is reachable from *u* if there exists a walk *u* = *v*_1_ → *v*_2_ → … → *v*_*ℓ*_ = *v* where *v*_*i*_ ∈ *V* (*i* = 1, …, *ℓ*). A digraph *G* is said to be strongly connected (diconnected) if every vertex is reachable from every other vertex. Clearly, the line digraph of a strongly connected digraph is also strongly connected. A cycle *C* ⊂ *V* in digraph *G* is a path *v*_1_ → *v*_2_ → … → *v*_*ℓ*_ → *v*_1_ where *v*_*i*_ ∈ *V*, *i* = 1, …, *ℓ*, are different nodes. Here *ℓ*(*C*) = *ℓ* is called the length of *C*. A digraph *G* is said to be aperiodic if the greatest common divisor of the lengths of its cycles is one. Formally, the period of *G* is defined as *per*(*G*) ≔ gcd{*ℓ* > 0: ∃*C* ⊂ *V* cycle such that *ℓ*(*C*) = *ℓ*}. Then, *G* is aperiodic if *per*(*G*) = 1. One can also see that the line digraph of an aperiodic digraph *G* is also aperiodic.

In a proper traffic, there are vehicles which leave or enter the city. To model these two possibilities *V* is augmented by a new ideal vertex 0 which denotes the world outside of the city. This approach is similar to that is applied for public transport in [60]. Let $\bar{V}≔V\cup \left\{0\right\}$. Then, additional directed edges which contains vertex 0 are also added to *E*. In this case, (*v*, 0) denotes that the vehicles can leave the city at vertex *v*, and (0, *v*) denotes that new vehicles can enter the city at vertex *v*, where *v* ∈ *V*. Let $\bar{E}$ denote the augmentation of *E* by directed edges for getting into and out of the city. Note that $\bar{E}$ does not contain the loop (0, 0). The augmentation of *G* is denoted by $\bar{G}=\left(\bar{V},\bar{E}\right)$ and it is called the closure of road network *G*. For $e=\left(v,w\right)\in \bar{E}$ we also use the notation *v* → *w*. Moreover, each aforementioned concept, e.g., strong connectivity, periodicity, line digraph, given for *G* can be extended for $\bar{G}$ in a natural way. Note that in the augmented line digraph $\text{L}\left(\bar{G}\right)=\left({\bar{V}}^{\prime },{\bar{E}}^{\prime }\right)$ the elements of the edge set ${\bar{E}}^{\prime }$ can be described by triplet (*u*, *v*, *w*), where $u,v,w\in \bar{V}$ such that *u* → *v* → *w* and if *v* = 0 then *u*, *w* ≠ 0 and if *u* or *w* is 0 then *v* ≠ 0 because triplets (0, 0, *v*), (*v*, 0, 0) and (0, 0, 0) are excluded from ${\bar{E}}^{\prime }$. One can easily see that if *G* is strongly connected (aperiodic) then its closure $\bar{G}$ (as well as $\text{L}(\bar{G})$) is also strongly connected (aperiodic). In the rest of this paper, it is assumed that the road network is closed, i.e., the vehicles can not get into and out of the road system of the city augmented with the ideal vertex 0. Moreover, for the sake of simplicity, only the first-order (primal) network is considered which is denoted by *G* as well.

We define vectors (functions) and matrices (operators or kernels) on *V* in the usual way. Let α:V→R denote a real function on *V* and let F(V,R) denote the set of real functions on *V*. We also use the notations ***α***(*v*) = ***α***_*v*_ for all *v* ∈ *V* and ***α*** = (***α***_*v*_)_*v*∈*V*_. F=F(V,R) is a finite dimensional vector space with the usual inner (dot) product. A T:V×V→R real function is called matrix, operator or kernel on *V* and induces a linear operator on F(V,R) in the usual way. Moreover, we write *T*(***α***) = *T**α*** as a matrix-vector product. If the support of *T* (the set {(*u*, *v*)|*u*, *v* ∈ *V*: *t*_*uv*_ ≠ 0} in *V* × *V*) is a subset of *E* (*E* ∪ *S*) then *T* is called *G*-subordinated in strong (weak) sense.

An example for matrix on *V* is the adjacency matrix *A* = (*a*_*uv*_)_*u*,*v*∈*V*_ of the digraph *G* where *a*_*uv*_ = 1 if and only if (*u*, *v*) ∈ *E* and 0 otherwise. Clearly, the support of *A* is *E*, i.e., *A* is a *G*-subordinated matrix in strong sense (*a*_*vv*_ = 0 for all *v* ∈ *V*). It is known that *G* is strongly connected if and only if there is a positive integer *k* such that the matrix *I* + *A* + … + *A*^*k*^ is positive, i.e., all the entries of this matrix are positive. The indegree and outdegree of a vertex *v* can be expressed by the adjacency matrix as $deg^{-}\left(v\right)=\sum_{u\in V}a_{uv}$ and *deg*^+^(*v*) = ∑_*u*∈*V*_ *a*_*vu*_. Introduce the vectors $d^{-}≔{\left(deg^{-}\left(v\right)\right)}_{v\in V}$ and ***d***^+^ ≔ (*deg*^+^(*v*))_*v*∈*V*_. Then, we have $d^{-}=A^{T}1$ and ***d***^+^ = *A* **1** where **1** ≔ (1)_*v*∈*V*_ is the constant unit function. It is well known that the adjacency matrix *A* of an aperiodic, strongly connected digraph *G* is primitive, i.e., irreducible and has only one eigenvalue of maximum modulus. Primitivity is equivalent to the following quasi-positivity: there exists k∈N such that the matrix *A*^*k*^ > 0, see Section 8.5 in [61].

### Probability distributions and Markov kernels on road networks

In this section, we summarize some basic concepts and results of the theory of finite Markov chains with their interpretations and consequences for traffic flow modeling. Some textbooks on this field are [62] and [63].

A probability distribution (p.d.) on *V* is the vector ***π*** ≔ (*π*_*v*_)_*v*∈*V*_ where ***π***_*v*_ ≥ 0 for all *v* ∈ *V* and ∑_*v*∈*V*_ *π*_*v*_ = 1. That is a p.d. ***π*** is a normalized $V\to R_{+}$ function. We can think of ***π***_*v*_ as the proportion of the number of vehicles which drive through the crossing *v* with respect to the whole number of vehicles in the city at a fixed time period. A Markov kernel or transition probability matrix on *V* is defined as a real kernel *P* ≔ (*p*_*uv*_)_*u*,*v*∈*V*_ such that *p*_*uv*_ ≥ 0 for all *u*, *v* ∈ *V* and ∑_*v*∈*V*_ *p*_*uv*_ = 1 for all *u* ∈ *V*, i.e., ***p***_*u*_ ≔ (*p*_*uv*_)_*v*∈*V*_ is a p.d. on *V* for all *u* ∈ *V*. The quantity *p*_*uv*_ ∈ [0, 1] is called the transition probability from vertex *u* to vertex *v*. The kernel *P* is said to be *G*-subordinated if *p*_*uv*_ > 0 for a pair *u*, *v* ∈ *V* implies (*u*, *v*) ∈ *E* or *u* = *v*, i.e., *P* as a matrix on *V* is *G*-subordinated in the weak sense. It is well known, see [64], that for a Markov kernel *P* on *V*, an associated digraph *G*_*P*_ = (*V*, *E*_*P*_) can be introduced in the following way: for a pair *u*, *v* ∈ *V* (where the case *u* = *v* is also allowed) (*u*, *v*) ∈ *E*_*P*_ if and only if *p*_*uv*_ > 0. Thus, *P* is *G*-subordinated if and only if *E*_*P*_ ⊆ *E* ∪ *S*, i.e., *G*_*P*_ is the subgraph of the digraph *G* extended with its loops *S*. In other words, a *G*-subordinated Markov kernel *P* is a stochastic matrix on *V* with support *E* ∪ *S*. Then, the sum condition for a *G*-subordinated Markov kernel *P* can be rewritten as:

$$\begin{array}{c} \sum_{w:v\to w}p_{vw}+p_{vv}=1,\;v\in V. \end{array}$$ (1)

(Note that *p*_00_ = 0).

A p.d. ***π*** on *V* is a stationary distribution (s.d.) of the kernel *P* if ∑_*u*∈*V*_ *π*_*u*_ *p*_*uv*_ = ***π***_*v*_ for all *v* ∈ *V*. For a *G*-subordinated Markov kernel *P* this formula, the so-called global balance equation, can be expressed as:

$$\begin{array}{c} \sum_{u:u\to v}\pi_{u}p_{uv}+\pi_{v}p_{vv}=\pi_{v},\;v\in V. \end{array}$$ (2)

**Fig 3:**
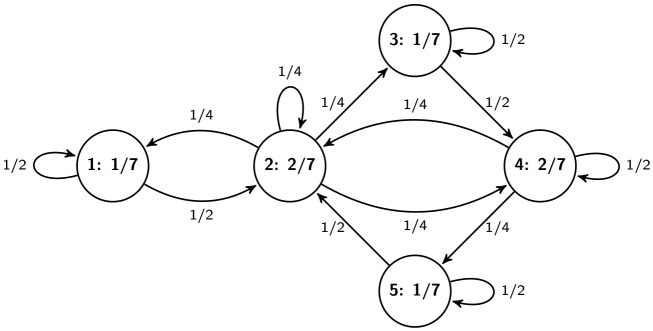
A Markov kernel (on edges) with its stationary distribution (on vertices) on the road network in Fig 1.

Fig 3 presents a Markov kernel with its s.d. on the road network in Fig 1.

The stationary distribution can be derived by solving the linear Eq (2) numerically. Since the state space (the road network) is finite there exists at least one stationary distribution. However, in some cases, the stationary distribution is not uniquely defined by these equations.

We show that there is a direct connection between the uniqueness of s.d. of a Markov kernel *P* on *V* and the strongly connected property of the physical road network *G* if the Markov and graph structures are compatible with each other. The Markov kernel *P* on *V* is called *G*-compatible if, for any *u*, *v* ∈ *V* such that *u* ≠ *v*, *p*_*uv*_ > 0 if and only if (*u*, *v*) ∈ *E*. Note that the *G*-compatibility implies the weak *G*-subordination for a Markov kernel *P*, however the converse is not true.

Clearly, if *P* is *G*-compatible then the strong connectivity of *G* implies that the associated graph *G*_*P*_ to the Markov kernel *P* is also strongly connected. In this case, the Markov kernel (the transition matrix) *P* is called irreducible. Thus, by Theorem 1 in [64], see also Theorem 3.1 and 3.3 in Chapter 3 of [63] the following theorem holds.

**Theorem 1**. If a road network *G* is strongly connected then there is a unique stationary distribution ***π*** to any *G*-compatible Markov kernel *P*. Moreover, this distribution satisfies *π*_*v*_ > 0 for all *v* ∈ *V*.

The main consequence of this theorem is that, in case of any physical road network augmented by the ideal vertex 0, all of the Markov kernels defined on the road network that has positive transition probability on all roads have unique stationary distribution. Thus, it is reasonable to suppose that a real traffic which follows a Markovian dynamic has a local unique stationary distribution in a short time period that can be explored by observing the traffic.

### Markov random walk and Markov traffic on road networks

Let (Ω,A,P) be a probability space. Then a *V*-valued random variable (r.v.) is a *X*: Ω → *V* measurable function, i.e., $X^{-1}\left(v\right)\in A$ for all *v* ∈ *V*. In this case, *X* is a random function on the set *V* of vertices. For example, *X* can be the random position of a vehicle on the road network *G*, where the position refers to the actual vertex which the vehicle belongs to. Then, $P(X^{-1}\left(v\right))=P\left(X=v\right)$ denotes the probability that a vehicle is at the vertex *v* ∈ *V*. By $\pi_{X}\left(v\right)≔P\left(X=v\right)$, *v* ∈ *V*, a r.v. *X* induces a p.d. ***π***_*X*_ on *V*.

A sequence ${\left\{X_{t}\right\}}_{t\in Z_{+}}$ ($Z_{+}=\left\{0,1,2,\ldots \right\}$) of *V*-valued r.v.’s is a Markov chain on the state space *V* if the Markov property holds:

$$\begin{array}{c} \begin{array}{c} P\left(X_{t}=v_{t}|X_{t-1}=v_{t-1},\ldots ,X_{0}=v_{0}\right)=P\left(X_{t}=v_{t}|X_{t-1}=v_{t-1}\right) \end{array} \end{array}$$

for all t∈N, *v*_0_, …, *v*_*t*_ ∈ *V*. If *X*, *X*′ are *V*-valued r.v.’s then for the conditional distribution *P* = (*p*_*vv*′_)_*v*,*v*′∈*V*_, $p_{vv^{\prime }}≔P\left(X=v|X^{\prime }=v^{\prime }\right)$, *v*, *v*′ ∈ *V*, we shall also use the notation *X*|*X*′. Clearly, *X*|*X*′ is a Markov kernel on *V*.

The main concepts of this paper are the Markov random walk and the Markov traffic defined in the following way.

**Definition 2**. Let the road network *G* be strongly connected and let *P* be a *G*-compatible Markov kernel on *V* with unique s.d. ***π***. Moreover, let ${\left\{X_{t}\right\}}_{t\in Z_{+}}$ be a Markov chain on *V* such that $\pi_{X_{0}}=\pi$ and *X*_*t*_|*X*_*t*−1_ ∼ *P* for all t∈N. Then, ${\left\{X_{t}\right\}}_{t\in Z_{+}}$ is called **Markov random walk** on the road network *G* with Markov kernel *P*.

The set $\left\{{\left\{X_{t}^{i}\right\}}_{t\in Z_{+}},i=1,\ldots ,k\right\}$ of *k* (k∈N) mutually independent Markov random walks on *G* with Markov kernel *P* is called a **Markov traffic** of size *k* and it is parametrized by the quadruple (*G*, *P*, ***π***, *k*).

The s.d. ***π*** of ${\left\{X_{t}\right\}}_{t\in Z_{+}}$ can be considered as a categorical distribution (generalized Bernoulli distribution) on F by formula $\pi \left(f\right)=\prod_{v\in V}\pi_{v}^{f_{v}}$ where f∈F is an indicator function, i.e., *f*_*v*_ = 1 for a fix *v* ∈ *V* and 0 otherwise, see page 75 in [65]. Since the Markov traffic $\left\{{\left\{X_{t}^{i}\right\}}_{t\in Z_{+}},i=1,\ldots ,k\right\}$ consists of *k* mutually independent Markov random walks its s.d. becomes the *k*-fold convolution π^*k^ on F, where * denotes the convolution. The p.d. π^*k^ fulfills

$$\begin{array}{c} \pi^{*k}\left(f\right)≔k!\prod_{v\in V}\frac{\pi_{v}^{f_{v}}}{f_{v}!} \end{array}$$ (3)

where f∈F is non-negative integer valued and satisfies the constraint ∑_*v*∈*V*_ *f*_*v*_ = *k*. In this case, ***f*** can be considered as traffic configuration where *f*_*v*_ counts the vehicles at vertex *v*. In fact, π^*k^ corresponds to the multinomial distribution with parameters *k* and ***π***, see Chapter 35 in [66]. Moreover, the r.v. $Y_{t}^{v}≔\sum_{i=1}^{k}I\left(X_{t}^{i}=v\right)$ follows the binomial distribution with parameters *k* and ***π***_*v*_ for all $t\in Z_{+}$ and *v* ∈ *V*, respectively. Note that the process ${\left\{Y_{t}^{v}\right\}}_{t\in Z_{+}}$ denotes the (random) number of vehicles at vertex *v* in the Markov traffic.

A similar model to Markov random walk is proposed in [67] where binary-coded edge-valued r.v.’s are considered (as dual view) instead of our vertex-valued r.v.’s (as primal view). Note that if *P* is the uniform Markov kernel on *G* then we obtain the standard random walk of the graph theory, see the survey [68].

A Markov random walk is an individual Markov traffic with *k* = 1 in the sense that it describes the movement of a random vehicle which follows the stochastic rules defined by the Markov kernel. On the other hand, the Markov traffic provides a mathematical model for describing the traffic of *k* vehicles on a road network. Note that the independence assumption seems reasonable and not too strong because the vehicle controls are working separately form each other. For a pair *u*, *v* ∈ *V* the notation *u* ⇒ *v* will mean that (*u*, *v*) ∈ *E* ∪ *S*, i.e., either *u* → *v* or *u* = *v*. If *X*_1_, *X*_2_ are random functions on *V* then *X*_1_ ⇒ *X*_2_ means that the two-dimensional distribution of (*X*_1_, *X*_2_) is concentrated on *E* ∪ *S*. Clearly, for any Markov random walk ${\left\{X_{t}\right\}}_{t\in Z_{+}}$ we have *X*_*t*_ ⇒ *X*_*t*+1_ ⇒ … ⇒ *X*_*t*+*n*_ for all *t* and n∈N. One can also call ${\left\{X_{t}\right\}}_{t\in Z_{+}}$ as a first-order random walk on the road network where a vehicle moves from vertex *u* to vertex *v* with probability *p*_*uv*_. The second-order Markov random walk (traffic) on the line road network, where the vehicles move from edge to edge, can also be defined similarly, see [58].

Using the concept of two-dimensional s.d. a Markov traffic can be reparametrized in the following way. Introduce the two-dimensional distribution *Q* = (*q*_*uv*_) on *V* × *V* as *q*_*uv*_ ≔ ***π***_*u*_ *p*_*uv*_, *u*, *v* ∈ *V*. Then, *Q* is a two-dimensional s.d. on *G* in the following sense:

**Definition 3**. A matrix *Q* = (*q*_*uv*_)_*u*,*v*∈*V*_ is called **two-dimensional stationary distribution** on *G* if (i) *q*_*uv*_ ≥ 0 for all *u*, *v* ∈ *V* and *q*_*uv*_ = 0 for all *u*, *v* ∈ *V* such that (*u*, *v*) ∉ *E* ∪ *S* (i.e., *Q* is weakly *G*-subordinated); (ii) ∑_*u*,*v*∈*V*_ *q*_*uv*_ = 1 (i.e., *Q* is a normalized matrix on *V*); and (iii) ∑_*v*∈*V*_ *q*_*uv*_ = ∑_*v*∈*V*_ *q*_*vu*_ for all *u* ∈ *V* (i.e., *Q* has equidistributed marginals).

A two-dimensional s.d. *Q* on *G* is called (strictly) positive if *q*_*uv*_ > 0 for all *u*, *v* ∈ *V* such that (*u* ⇒ *v*) *u* → *v*.

**Fig 4:**
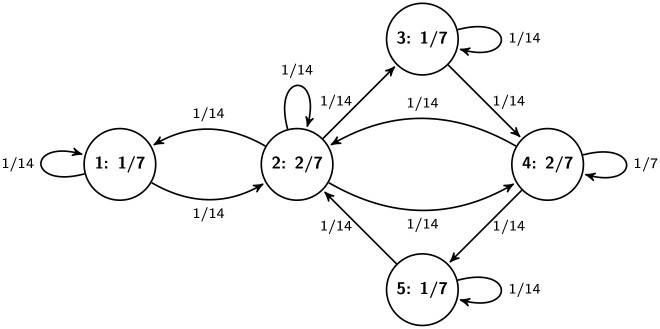
The two-dimensional s.d. (on edges) with its equidistributed marginals (on vertices) on the road network in Fig 1 for the Markov kernel in Fig 3. One can easily check that the sums of probabilities written on the edges in and out each vertex are equal, respectively.

Property (iii) states that the two (row-wise and column-wise) marginal distributions of a two-dimensional s.d. on *G* coincide with each other. Clearly, for a Markov traffic, *Q* defined above is a positive two-dimensional distribution on *G*. *Q* can also be considered as a p.d. on the state space *E* ∪ *S*, i.e., if we extend the set *V*′ of vertices of L(*G*) as *V*′ = *E* ∪ *S*, on the line digraph. Thus, *Q* can be interpreted as the distribution of the vehicles on the edges of the road network, i.e., on the line digraph, see formula (11) in [21]. The distribution *Q* can also be visualized on the edges, see, Fig 4 for the simple example in Figs 1 and 11 in case of the Porto example discussed later. However, the converse of this statement is not true because there is p.d. on the line digraph which does not satisfy (iii). If ${\left\{X_{t}\right\}}_{t\in Z_{+}}$ is a Markov random walk then the two dimensional distribution of any consecutive pair (*X*_*t*_, *X*_*t*+1_), $t\in Z_{+}$, corresponds with *Q*.

Denote by Q the set of two-dimensional s.d. on *G*. One can easily see that Q is closed with respect to the affine combination. Namely, if $Q_{1},Q_{2}\in Q$ then $\lambda Q_{1}+\left(1-\lambda \right)Q_{2}\in Q$ for all λ ∈ [0, 1].

Conversely, for a positive Q∈Q, let us define

$$\begin{array}{c} \begin{array}{cc} \pi_{u}≔ & \sum_{v\in V}q_{uv}=\sum_{v\in V}q_{vu},\;u\in V,\;\\ p_{uv}≔ & \frac{q_{uv}}{\pi_{u}},\;u,v\in V. \end{array} \end{array}$$ (4)

Then, *P* = (*p*_*uv*_) defines a *G*-compatible Markov kernel with s.d. ***π*** = (*π*_*u*_) on *G*. Thus, a Markov traffic defined by the quadruple (*G*, *P*, ***π***, *k*) can be introduced by an equivalent way through the triplet (*G*, *Q*, *k*). However, it will turn out later that, from a statistical point of view, the parameter matrix *Q* can be estimated in a computationally more efficient way than the pair of transition matrix *P* and its s.d. ***π***.

### Ergodicity of Markov traffic

The simulation method proposed in this paper is based on the ergodicity of Markov traffic which follows from the ergodic theory of finite Markov chains.

Let ***π***_0_ be an initial distribution on *V* and define the *n*-th absolute p.d. ***π***_*n*_ by the recursion $\pi_{n}^{⊤}=\pi_{n-1}^{⊤}P$, n∈N. Clearly, $\pi_{n}^{⊤}=\pi_{0}^{⊤}P^{n}$, where the product of two Markov kernels *P* and *Q* on *V* is defined as (*PQ*)_*uw*_ ≔ ∑_*v*∈*V*_ *p*_*uv*_ *q*_*vw*_, *u*, *w* ∈ *V*. If *G* is strongly connected and we start from the unique s.d. ***π***_0_ = ***π*** of *P*, see Theorem 1, then $\pi_{n}^{⊤}=\pi^{⊤}P^{n}=\pi^{⊤}$ for all n∈N. Thus, in this case, ***π***_*n*_ → ***π*** as *n* → ∞. However, in general, the sequence ${\left(\pi_{n}\right)}_{n\in N}$ does not converge to the s.d. for all initial distribution ***π***_0_ even if the s.d. is unique. However, if we consider the average of the *n*-th absolute p.d.’s in time, we have the convergence to the unique s.d. The Markov kernel which satisfies this property is called ergodic.

The following result is based on Theorem 4.1 in Chapter 3 of [63]. If a road network *G* is strongly connected then any *G*-compatible Markov kernel *P* is ergodic and the average Markov kernel *A*_*n*_ converges, i.e.,

$$\begin{array}{c} A_{n}≔{\left(n+1\right)}^{-1}\left(I+P+\ldots +P^{n}\right)\to \Pi ≔1\pi^{⊤} \end{array}$$

as *n* → ∞, where ***π*** is the unique s.d. of *P*. Moreover, the limiting probabilities of the time averages of the absolute p.d.’s satisfy

$$\begin{array}{c} {\left(n+1\right)}^{-1}\left(\pi_{0}+\pi_{1}+\ldots +\pi_{n}\right)\to \pi \end{array}$$ (5)

as *n* → ∞ for all initial p.d. ***π***_0_.

In applications, along absolute p.d.’s, we may also be interested in some functionals of these distributions, e.g., the number of vehicles in a region of the road network. Define the functional ***f***(***π***) ≔ ∑_*v*∈*V*_ *f*_*v*_ *π*_*v*_ of p.d. ***π***, where f∈F. Then, (5) can be extended that $n^{-1}\sum_{i=1}^{n}F\left(\pi_{i}\right)\to F\left(\pi \right)$ as *n* → ∞, see Theorem 4.1 of [63].

Instead of time averages, in order to achieve the convergence of *n*-th absolute p.d.’s we need the additional assumption of aperodicity for *G*, see Theorem 2.1 in Chapter 4 of [63]. If *G* is an aperiodic, strongly connected road network and *P* is a *G*-compatible Markov kernel on it, then the sequence of Markov kernels *P*^*n*^, n∈N, converges to the limiting Markov kernel *Π*. Moreover, the limit of the sequence of *n*-th absolute p.d. ***π***_*n*_ is the unique s.d. ***π*** to the Markov kernel *P* which is independent of the initial p.d. ***π***_0_. For any functional *F* we also have that ***F***(*π*_*n*_) converges to ***F***(*π*) as *n* → ∞ on an aperiodic, strongly connected road network.

The ergodicity of Markov traffic with any *G*-compatible Markov kernel is derived in the same way. Let $\pi_{0}^{i}$ and $\pi_{n}^{i}$ denote the initial distribution and *n*-th absolute p.d., respectively, for the *i*th vehicle, *i* = 1, …, *k*. Then, the ergodic property of Markov traffic, similarly to (5), can be formulated as

$$\begin{array}{c} {\left(n+1\right)}^{-1}\sum_{i=0}^{n}\left(\pi_{i}^{1}*\ldots *\pi_{i}^{k}\right)\to \pi^{*k} \end{array}$$ (6)

if *n* → ∞, i.e., the time average of the probability of complex traffic events converges to a constant which corresponds to their stationary probability. Since the mean of the multinomial distribution with parameters *k* and ***π*** is calculated as *k**π*** one can see that the probabilities in s.d. ***π*** on *V* can be unfolded by the limit of state-space and time averages as

$$\begin{array}{c} {\left(\left(n+1\right)k\right)}^{-1}\sum_{i=0}^{n}\sum_{f\in F}f_{v}\cdot \left(\pi_{i}^{1}*\ldots *\pi_{i}^{n}\right)\left(f\right)\to \pi_{v} \end{array}$$ (7)

as *n* → ∞ for all *v* ∈ *V*. Note that the left hand side of (7) is the average of the number of vehicles at vertex *v* in time divided by the size of the traffic (*k*).

The convergence results (6) and (7) guarantee that the unique s.d. of a *G*-compatible Markov kernel can be approximated and thus explored by long run behavior of the traffic flow on the road network. A visualization of the convergence of Markov traffic simulation to its s.d. is presented in Fig 10.

### Trajectories from public datasets

For our experiments, we needed a dataset of real-life traffic trajectory data. In our terminology, a trajectory is a sequence of data that provides information about the path of a vehicle moving from a start to an end point, associating geographic coordinates with timestamps. We required a dataset that satisfies the following criteria:

1. Contains complete trajectories, i.e., the availability of only the start and end points is not sufficient, intermediate trajectory points must also be available.
2. The trajectory points must be sampled at a high enough frequency, so that the distance between consecutive points should not be too large, (e.g., an average distance of the order of 10 meters is acceptable, but an average distance of the order of 100 meters is definitely not).
3. The dataset is sufficiently large. It should cover a long enough period of time, preferably uniformly. The number of trajectories per day should be of the order of thousands.
4. Trajectories should cover a relatively small geographic area, e.g., a city or a district.
5. Vehicles should not follow a fixed route, e.g., public transport bus trajectories are not suitable.
6. Publicly available for research purposes.

**Fig 5:**
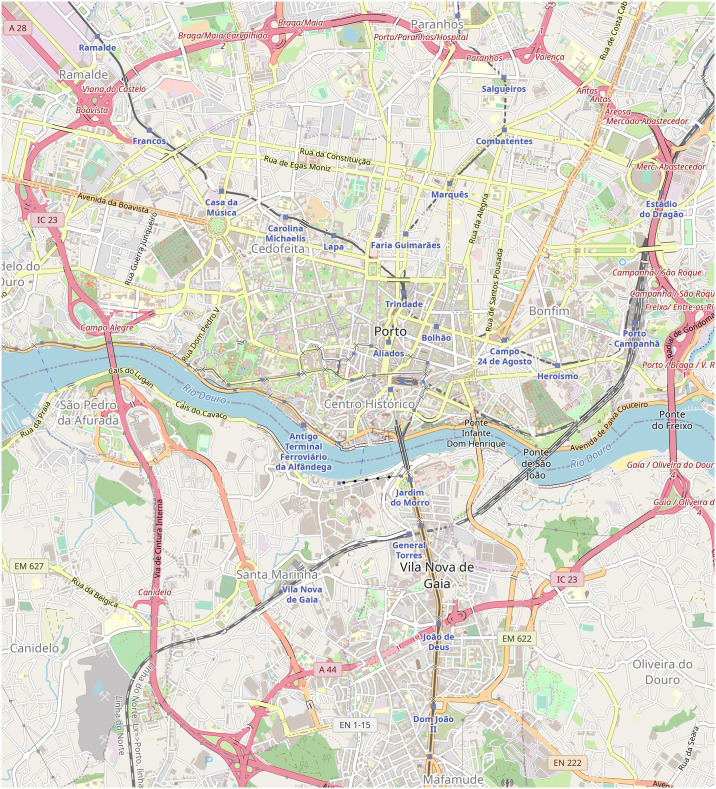
The map of the observed area. The graph created from the OSM data has 33,961 nodes, 53,126 edges, and covers a total of 857.26 km of road. The size of the area is about 43.68 km^2^. (Base map and data from OpenStreetMap and OpenStreetMap Foundation. Reprinted from OpenStreetMap under a CC BY license, with permission from OpenStreetMap, original copyright 2020. ©OpenStreetMap contributors).

**Fig 6:**
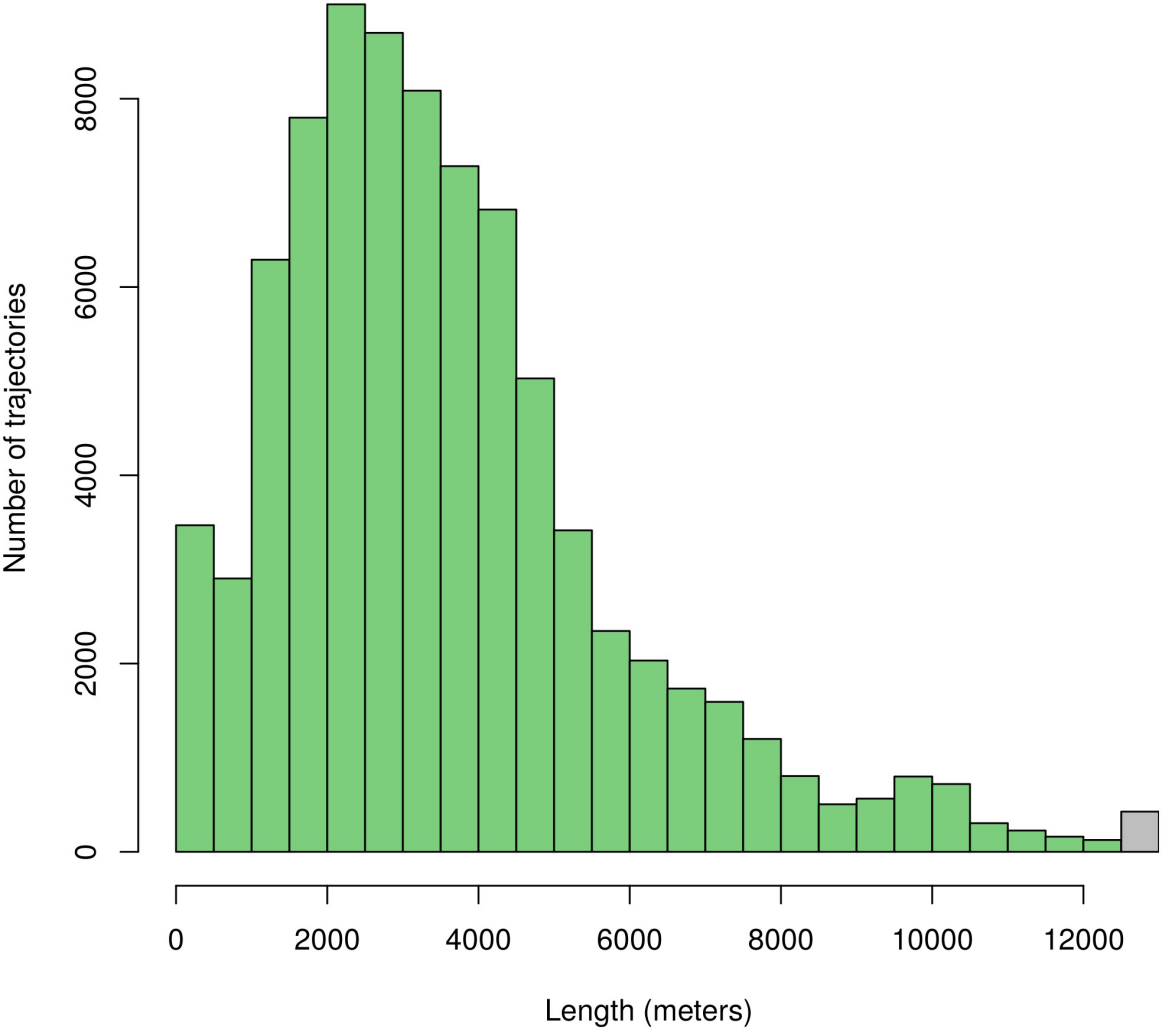
Histogram of trajectory lengths. The rightmost bar represents trajectories longer than 12,500 meters. The average trajectory length is 3,628.93 meters.

**Fig 7:**
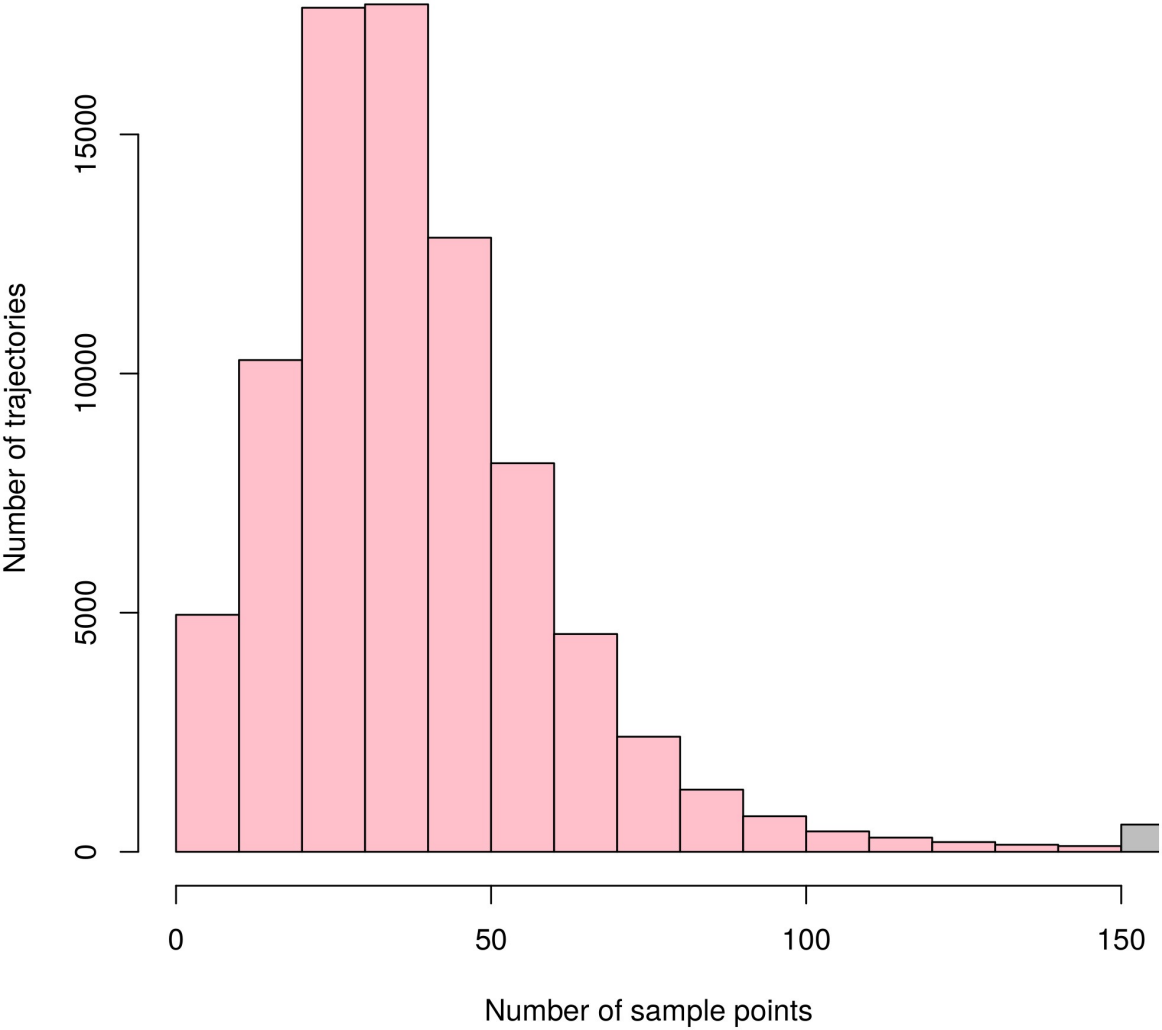
Histogram of number of sample points per trajectories. The rightmost bar represents trajectories with more than 200 sample points. On the average, a trajectory consists of 40 sample points and takes 10 minutes.

**Fig 8:**
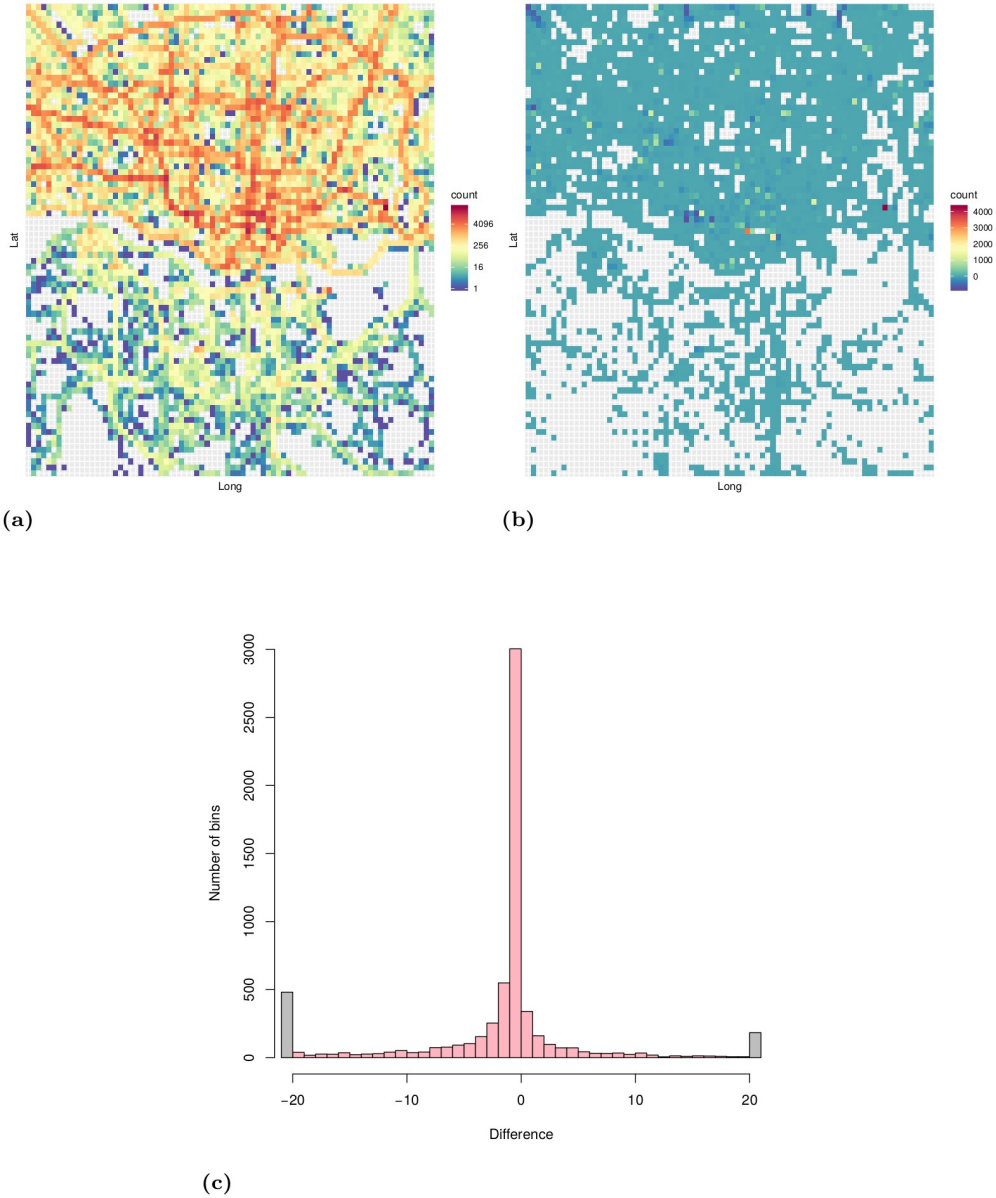
Distribution of trajectory points of the filtered dataset. a: Distribution of all trajectory points shown in a 2D histogram (number of bins: 80 × 80). b: Difference of trajectory starting and endpoints shown in a 2D histogram (number of bins: 80 × 80). The color of each bin represents the number of trajectory starting points minus the number of trajectory endpoints that fall in that bin. c: Histogram of the difference of trajectory starting and endpoints.

These requirements were satisfied by the Taxi Trajectory Prediction (TTP) dataset from Kaggle. The dataset covers a period of one year from July 1, 2013 to June 30, 2014. It is split into a training and a test set, the former contains 1,710,670 trajectories, and the latter contains 320. The trajectories were collected in the city of Porto, Portugal, with a sampling rate of 15 seconds. First, we created a subset of the dataset, filtered to coordinates between W8.6518, W8.5771, N41.1129, N41.1756, see Fig 5. The data samples’ features that were not relevant to the research, such as origin of call, identifiers for individual taxi or customers, and type of day (i.e. workday, weekend, holiday) were omitted. The processed format included the time of departure, both as a timestamp and as distinct date attributes, the length of the trajectory, and the points of the trajectory, represented as a list of GPS coordinates. Some data samples contained incomplete trajectories, these were discarded. Because of the properties of the proposed simulation model, the data was filtered to include only those samples that had a time of departure between 8-9 am. As a result, 82,345 trajectories remained. Although the length of trajectories had a wide range (the longest has 2,324 sample points), long trips were rare. Fig 6 shows the distribution of the length of trajectories. Most routes were around a length of 41 sample points, and routes with over 150 points were less than 1% of the dataset, see Fig 7. The distribution of the trajectory points (all, difference of start and end points, histogram of the difference) is shown in Fig 8. The descriptive statistics of the dataset is shown in Table 1.

**Table 1:** Descriptive statistics of lengths of trajectories. (82,345 total trajectories).

Name of statistics	Dist in points	Dist in meters
Mean	39.53	3,628.93
Median	35	3,176.786
Mode	34	0
Standard Deviation	31.64	2,408.93
Kurtosis	473.28	9.9
Skewness	12.11	1.78
Minimum	2	0
Maximum	2,324	61,055.58

### Building graphs from OpenStreetMap data

OpenStreetMap (OSM) is a community project to build a free map of the world to which anyone can contribute. Data is available under the Open Data Commons Open Database License (ODbL). The representation and storing of map data is based on a simple but powerful model, that uses only three modeling primitives, namely, nodes, ways, and relations: 1. A node represents a geographical entity with GPS coordinates. 2. A way is an ordered list of at least two nodes. 3. A relation is an ordered list of nodes, ways, and/or relations. All of these modeling elements can have associated key-value pairs called tags that describe and refine the meaning of the element to which they belong. Users can export map data at the OSM web site manually, selecting a rectangular region of the map. Alternatively, map data can be extracted via web services, see http://wiki.openstreetmap.org/wiki/API. OSM uses two formats for exporting map data, namely OSM XML and PBF. Software libraries for parsing and working with OSM data are available for several programming languages, see https://wiki.openstreetmap.org/wiki/Frameworks.

We started our processing by building a graph from the OSM map of Porto, with the same bounding box as the filtered dataset. Specific nodes of the OSM file become the nodes in the graph. Because we only need those nodes that can be reached via vehicles, we had to filter the OSM file and collect only specific types of way nodes. In the OSM file, a way is a sequence of OSM nodes, so naturally, the nodes of ways become nodes in the graph. For every node we store the node’s OSM ID, and its coordinates. We also insert an edge into the graph between every nodes in way. The weight of an edge is given by the squared distance between the nodes, which we calculate from the OSM file’s data. We used pyosmium library for processing the OSM files and the NetworkX Python library for building the graph.

After building the graph we process the list of trajectories. Because the trajectories are given in GPS coordinates, we first have to translate those coordinates into OSM node IDs. For every coordinate in a trajectory, we search for the closest way node’s coordinates in the built graph, so the result nodes have the same domain as the built graph’s nodes. Obviously, the original trajectories made up of GPS coordinates does not have the same scaling as the OSM map. The coordinates in the trajectory are sampled in regular, but larger time intervals than the OSM, so they are not aligned. In order to match a trajectory to a way in our graph, we had to perform an interpolation on the result list of node IDs, so we ran a Dijkstra’s shortest path algorithm on our graph between every node IDs for every trajectory. Because the OSM database contains errors, it can happen that in real life a route exists between two given places, but in the OSM database, there are no existing routes between those nodes that are representing the given places. In this case, we cut the faulty trajectories into pieces. The result of this process is an aperiodic strongly connected road network augmented by the ideal vertex 0, with a set of trajectories on the road network.

## Statistical inference for Markov traffic using mobile sensors

The statistical analysis of a traffic systems described by the Markov traffic model means the estimation of the quadruple (*G*, *P*, ***π***, *k*) or the triplet (*G*, *Q*, *k*) using observed data. To estimate *G* we have to explore the road system under study by identifying the set *V* of vertices and the set *E* of directed road segments. Fortunately, this exploring has already been done by a few organizations, see, e.g., the Google Maps and the OpenStreetMap. However, we should note that, in case of GPS-based trajectory data, we have to fit the data to the applied map system which is not an evident task at all. In the present paper, we propose a method for estimating the two-dimensional stationary distribution *Q* immediately instead of the pair (*P*, ***π***) of a transition matrix and its stationary distribution using mobile sensor data which may be gathered by vehicles, passengers etc. In this case, we have trajectories data which consists of the sequences of consecutive vertices, like in the TTP dataset. By (4), the estimators for *P* and ***π*** can be easily derived from an estimator of *Q*. Finally, it is supposed that the size *k* of the traffic is known.

Suppose that, for a Markov traffic, we observed a random sample of trajectories {*X*^*i*^}, *i* = 1, …, *k*, of size *k* defined by $X_{1}^{i}\Rightarrow X_{2}^{i}\Rightarrow \ldots \Rightarrow X_{n_{i}}^{i}$, *i* = 1, …, *k*, where *n*_*i*_ denotes the length of the *i*-th trajectory. Let *n* ≔ *n*_1_ + … + *n*_*k*_ be the total sample size. Define the total two-dimensional consecutive empirical frequencies as:

$$\begin{array}{c} n_{uv}≔\sum_{i=1}^{k}n_{uv}^{i}, \end{array}$$ (8)

*u*, *v* ∈ *V*, where the trajectory-wise two-dimensional consecutive empirical frequencies, *i* = 1, …, *k*, are defined as

$$\begin{array}{c} n_{uv}^{i}≔\sum_{j=1}^{n_{i}-1}I\left(X_{j}^{i}=u,X_{j+1}^{i}=v\right), \end{array}$$

*u*, *v* ∈ *V*. Plainly, $n_{uv}^{i}$ denotes the number of consecutive (*u*, *v*) (*u*, *v* ∈ *V*) pairs in the *i*-th trajectory. One can see that since {*X*^*i*^} is a proper Markov random walk we have $n_{uv}^{i}=0$ for all (*u*, *v*)∉*E* ∪ *S*. Thus, the support of the two-dimensional frequency matrices *N* ≔ (*n*_*uv*_)_*u*,*v*∈*V*_, $N_{i}≔{\left(n_{uv}^{i}\right)}_{u,v\in V}$, *i* = 1, …, *k*, is a subset of *E* ∪ *S*, i.e., they are weakly *G*-subordinated matrices. Clearly, $N=\sum_{i=1}^{k}N_{i}$ and we have

$$\begin{array}{c} \sum_{u,v:u\Rightarrow v}n_{uv}=n-k, \end{array}$$ (9)

where *n* − *k* is the corrected sample size. Introduce

$$\begin{array}{c} s_{v}≔\sum_{i=1}^{k}I\left(X_{1}^{i}=v\right),\;e_{v}≔\sum_{i=1}^{k}I\left(X_{n_{i}}^{i}=v\right), \end{array}$$

*v* ∈ *V*, i.e., *s*_*v*_ denotes the number of trajectories which start at vertex *v* and *e*_*v*_ denotes the number of trajectories which terminate at vertex *v*, respectively. Denote the one-dimensional marginal frequencies of *N* by *n*_*v*+_ ≔ ∑_*u*∈*V*_ *n*_*vu*_ and *n*_+*v*_ ≔ ∑_*u*∈*V*_ *n*_*uv*_, *v* ∈ *V*. We obtain that

$$\begin{array}{c} n_{v+}+e_{v}=n_{+v}+s_{v}=n_{v}≔\sum_{i=1}^{k}\sum_{j=1}^{n_{i}}I\left(X_{j}^{i}=v\right) \end{array}$$ (10)

for all *v* ∈ *V*, where *n*_*v*_ denotes the number of vertex *v* in all trajectories. Finally,

$$\begin{array}{c} \sum_{v\in V}s_{v}=\sum_{v\in V}e_{v}=k. \end{array}$$ (11)

Define the vectors ***s*** and ***e*** on *V* as ***s*** ≔ (*s*_*v*_)_*v*∈*V*_ and ***e*** ≔ (***e***_*v*_)_*v*∈*V*_, respectively. Then, (11) implies that **1**^⊤^(*e* − *s*) = 0, i.e., the vectors ***e*** − ***s*** and **1** are orthogonal.

The traditional maximum likelihood (ML) estimator ${\hat{P}}_{\text{ML}}$ of the transition matrix *P* is given by the maximization of the conditional loglikelihood

$$\begin{array}{c} \text{log}\;L=\sum_{u\Rightarrow v}n_{uv}\;\text{log}\;p_{uv} \end{array}$$

in parameters *p*_*uv*_, *u*, *v* ∈ *V* such that *u* ⇒ *v*, under the constraints *p*_*u*+_ = 1 for all *u* ∈ *V*. A solution of this constrained optimization problem is ${\hat{p}}_{uv}^{\text{ML}}=n_{uv}/n_{u+}$ for all *u*, *v* ∈ *V* if *n*_*u*+_ > 0 and ${\hat{p}}_{uv}^{\text{ML}}=\delta_{uv}$ if *n*_*u*+_ = 0 where *δ* denotes the Kronecker delta. The maximum likelihood estimator ${\hat{\pi }}_{\text{ML}}$ of the stationary distribution ***π*** is derived by the solution of the global balance equation $\pi^{⊤}=\pi^{⊤}{\hat{P}}_{\text{ML}}$ in ***π***. Thus, the maximum likelihood estimator ${\hat{Q}}_{\text{ML}}=\left({\hat{q}}_{uv}^{\text{ML}}\right)$ of *Q* is given by ${\hat{q}}_{uv}^{\text{ML}}={\hat{\pi }}_{u}^{\text{ML}}{\hat{p}}_{uv}^{\text{ML}}$, *u*, *v* ∈ *V*. In the sequel, a direct method is proposed for estimating the two-dimensional stationary distribution *Q*.

A naîve estimator for the two-dimensional stationary distribution *Q* based on the two-dimensional consecutive empirical frequency matrix *N* is ${\hat{Q}}_{\text{na}î\text{ve}}≔{\left(n-k\right)}^{-1}N$. Clearly, ${\hat{Q}}_{\text{na}î\text{ve}}$, as a non-negative matrix on *V*, satisfies the properties (i) and (ii) of Definition 3. However, the problem with this naîve estimator is that its row and column marginals are not necessarily equal, i.e., in general, it does not satisfy the asumption (iii) of Definition 3. Hence, we have to introduce a new estimator $\hat{Q}$ which belongs to Q and is optimal in some sense.

The optimality of the proposed estimator is defined by means of the least squares distance between matrices over *G*. Let *A* = (*a*_*uv*_)_*u*,*v*∈*V*_ and *B* = (*b*_*uv*_)_*u*,*v*∈*V*_ such that *a*_*uv*_ = *b*_*uv*_ = 0 for all *u*, *v* ∈ *V* where u⇏v, i.e., let *A* and *B* be weakly *G*-subordinated matrices. The distance between *A* and *B* is defined as

$$\begin{array}{c} {\left\|A-B\right\|}_{G}≔{\left(\sum_{u,v:u\Rightarrow v}{\left|a_{uv}-b_{uv}\right|}^{2}\right)}^{1/2}. \end{array}$$

In fact, ‖⋅‖_*G*_ is the Frobenius norm of the matrices of dimension |*V*| × |*V*| which vanish on the entries outside of *E* ∪ *S*.

To formulate the objective function for estimating the two-dimensional stationary distribution it is convenient to weaken the assumptions of Definition 3 by leaving the normalizing assumption (ii). In the sequel, let *M* = (*m*_*uv*_) denote a non-negative parameter matrix on *G* which satisfies assumptions (i) and (iii) of Definition 3, i.e., *M* is weakly *G*-subordinated and ∑_*v*∈*V*_ *m*_*uv*_ = ∑_*v*∈*V*_ *m*_*vu*_ for all *u* ∈ *V*. Then, one can easily derive a two-dimensional stationary distribution *Q* from *M* by its normalization defining as *Q* ≔ (**1**^⊤^ *M* **1**)^−1^ *M*.

Based on *k* number of trajectories, using the Frobenius norm, the optimality criterion is defined as the weighted sum of squared errors (SSE):

$$\begin{array}{c} \text{SSE}\left(M,w\;|\;N\right)≔\sum_{i=1}^{k}w_{i}^{-1}{\left\|N_{i}-w_{i}M\right\|}_{G}^{2}, \end{array}$$ (12)

where *M* is a non-negative parameter matrix satisfying assumptions (i) and (iii) of Definition 3, ***w*** = (*w*_*i*_)_*i*=1,…,*k*_ are non-negative unknown weights, with $\sum_{i=1}^{k}w_{i}=1$, and ***N*** ≔ (*N*_*i*_)_*i*=1,…,*k*_ denotes the data, where *N*_*i*_ is the two-dimensional consecutive empirical frequency matrix for the *i*th trajectory, see (8). The statistical inference for a Markov traffic means the minimization of the objective function SSE in its parameters *M* and ***w*** deriving the weighted least squares (WLS) estimators ${\hat{M}}_{\text{WLS}}$ and ${\hat{w}}_{\text{WLS}}$. Then, the WLS estimator of *Q* is defined as ${\hat{Q}}_{\text{WLS}}≔n_{\text{eff}}^{-1}{\hat{M}}_{\text{WLS}}$ where $n_{\text{eff}}≔\left(1^{⊤}{\hat{M}}_{\text{WLS}}1\right)$ is the so-called effective sample size. Here, $\hat{Q}$ can be interpreted as the estimated two-dimensional stationary distribution which describes the individual Markov traffic in time. On the other hand, *n*_eff_ gives the equivalent sample size related to the independent case which may be thought of as the information content of the observed data. Note that *n*_eff_ is not necessarily an integer and is different from *n* and *n* − *k*. Finally, *w*_*i*_ gives the importance of the *i*th trajectory in the sample. One can see that longer trajectory implies higher weight.

To formulate our result on WLS estimation of Markov traffic we need some basic facts on the spectral theory of directed graphs, see [69] for details. The symmetric unnormalized graph Laplacian matrix *L* of a digraph *G* is defined as

$$\begin{array}{c} L≔D-A-A^{⊤} \end{array}$$

where *A* denotes the adjacency matrix of *G* and *D* ≔ diag{***d***^+^ + ***d***^−^} is a diagonal matrix. Note that for the road graph *G*, since there is no loop, we have *l*_*vv*_ = *d*_*vv*_ = deg^+^(*v*) + deg^−^(*v*) for all *v* ∈ *V*. The main theorem of this paper is the following.

**Theorem 2**. There is a unique pair $\left({\hat{M}}_{\text{WLS}},{\hat{w}}_{\text{WLS}}\right)$ which minimizes the weighted sum of squared errors SSE defined in (12). These WLS estimators are derived as

$$\begin{array}{c} {\hat{w}}_{\text{WLS}}^{i}≔\frac{\|N_{i}{\|}_{G}}{\sum_{j=1}^{k}{\left\|N_{j}\right\|}_{G}}, \end{array}$$

*i* = 1, …, *k*, and

$$\begin{array}{c} {\hat{M}}_{\text{WLS}}≔N+\left(1\lambda^{⊤}-\lambda 1^{⊤}\right)\circ A, \end{array}$$

where λ∈F is called Lagrange vector and defined as a unique solution to the linear equation *L* **λ** = *s* − ***e*** which satisfies the constraint **1**^⊤^ **λ** = 0 (i.e., ∑_*v*∈*V*_ λ_*v*_ = 0) and ∘ denotes the entrywise (Hadamard) product of matrices.

Based on the previous theorem, by (9), the effective sample size is given as

$$\begin{array}{c} n_{\text{eff}}≔\left(n-k\right)+{\left(d^{-}-d^{+}\right)}^{⊤}\lambda , \end{array}$$ (13)

i.e., *n*_eff_ depends only on the graph structure of the road network, which is independent of the data, the traffic direction vector ***s*** − ***e***, and the corrected sample size. However, it does not depend on the data which are inside the trajectories.

The WLS estimators proposed above can be considered as a kind of composite (or quasi-) likelihood estimators for Markov chains, see [54]. The composite likelihood method is widely applied in complex statistical models when the full ML method can be difficult to apply or may not be robust enough. In our method, the objective function is based on pairwise marginal distributions, however, instead of formula (2) in [54], the quasi-likelihood function is a square function, the logarithm of the normal probability density with heteroscedastic variance which depends on the length of trajectories. The latter will be more clear by introducing the mean squared error (MSE) as

$$\begin{array}{c} \text{MSE}≔n_{\text{eff}}^{-1}\text{SSE}=\sum_{i=1}^{k}n_{\text{eff}}^{i}{\left\|{\left(n_{\text{eff}}^{i}\right)}^{-1}N_{i}-Q\right\|}_{G}^{2}, \end{array}$$ (14)

where $n_{\text{eff}}^{i}≔w_{i}n_{\text{eff}}$ denotes the effective sample size of the *i*th trajectory, *i* = 1, …, *k*. The parameters of the objective function MSE are the effective sample sizes $\left\{n_{\text{eff}}^{i}\right\}$ and the two-dimensional s.d. *Q*. The heuristic explanation of the need to use weights in formulas (12) and (14) is the following. By the Central Limit Theorem, for large *n*_*i*_, the trajectory-wise two-dimensional consecutive empirical frequency matrix *N*_*i*_ can be approximated as $N_{i}\approx n_{i}Q+n_{i}^{1/2}\xi_{i}$, where ***ξ***_*i*_ is a normally distributed random matrix on *V* for all *i* = 1, …, *k*, which is a heteroscedastic equation between the observed *N*_*i*_ and the parameter matrix *Q*. Hence, $\|N_{i}-n_{i}{Q\|}_{G}^{2}\approx n_{i}{\left\|\xi_{i}\right\|}_{G}^{2}$, where $\|\xi_{i}{\|}_{G}^{2}$, *i* = 1, …, *k*, are independent identically distributed r.v.’s. Thus, we have to normalize the trajectory-wise squared errors proportionally to their lengths, respectively, in order to get balanced error terms.

The estimation theory of finite Markov chains goes back for a long time, see [70]. In the traditional ML approach the estimators of the transition and stationary probabilities are derived by corresponding relative frequencies, respectively. However, these estimators have a few problems which imply that they can be applied with limited success for estimating the Markov traffic on a road network. Firstly, they are based on only one long trajectory (or realization). However, in a real traffic dataset there is a large number of relatively short trajectories, i.e., the set {*n*_*i*_, *i* = 1, …, *k*} are bounded, where *k* is large or tends to infinity. In our example, for the TTP dataset, the number of trajectories is above 80K with the mean length 40 and maximum length 2K, see Table 1. Secondly, they are asymptotic estimators in the sense that, for finite sample size, the estimated stationary distribution does not satisfy the global balance equation given by the estimated transition probability matrix. The global balance equation holds only asymptotically, i.e., when the sample size tends to infinity. In fact, the inaccuracy in the global balance equation is not too large, however, this little bias can cause significant discrepancy from the “true” stationary distribution in the simulation. Thirdly, the trajectories are biased during a short time period in the sense that they are starting from some parts of the road network and ending at other parts. For example, in the morning period the vehicles are moving from the residential districts to the business districts of the city and they are moving back in the afternoon period. In other words, the traffic has a definite direction on the road network. To demonstrate this behavior in the case of TTP dataset, Fig 8c shows the distribution of the elements of the traffic direction vector ***s*** − ***e*** while Fig 8b shows their spatial distribution. Neither distributions are concentrated around the zero. The known improvements of the ML estimators, e.g., by using the bootstrap, see [71], do not solve these problems. However, the WLS estimator of the two-dimensional stationary distribution proposed in this paper is able to handle all of these problems. The estimator ${\hat{Q}}_{\text{WLS}}$ is taking account of more than one trajectory with their length. It determines uniquely both the transition probability matrix and its stationary distribution by (4) which satisfy the global balance equation obviously. Finally, by taking account of the traffic direction vector in the estimator, it can correct the bias due to the unbalanced sampling of trajectories on the road network.

The fundamental statement of Theorem 2, as one of the main result of this paper, is that the estimator ${\hat{Q}}_{\text{WLS}}$ (or ${\hat{M}}_{\text{WLS}}$) consists of two parts: the first part is the naîve estimator for the distribution of the consecutive pairs in trajectories based on the empirical frequencies, while the second part is a correction term ensuring that ${\hat{Q}}_{\text{WLS}}$ (or ${\hat{M}}_{\text{WLS}}$) has equidistributed marginals. The second part also depends on two components. The first one is the Laplacian matrix of the road graph which depends only on the graph structure of the road network and independent from the trajectory data. The second one is the traffic direction vector which depends only on the trajectory data. Note that all sufficient statistics, namely the total two-dimensional consecutive empirical frequencies and starting and ending empirical frequencies, can be computed by counting, which is numerically very effective and can be executed even for big data.

The computationally intensive part of WLS estimator is the numerical solution of sparse linear equation system given for the Lagrange vector **λ** in Theorem 2. This can be performed in a numerically effective manner by the eigenvalues-eigenvectors decomposition of the symmetric unnormalized (or normalized) graph Laplacian matrix, see Proposition 1 and 2 in [72]. Remark that the first few significant eigenvalues and eigenvectors, being independent from the data, can be computed and stored in advance for a simulation program. Finally, one can also see that, similarly to the Google’s PageRank algorithm, see Chapter 15 in [73]), a linear recursion could be computationally more efficient in large-scale problems.

## Results

To evaluate the performance of the proposed WLS estimation method by comparing it to the traditional ML one discussed above, a simple simulation study was conducted at different sample sizes for small and medium road network. In the simulations, in order to mimic the real traffic, we tried to keep the length of trajectories low and the number of trajectories high compared to the size of the road network, similarly to the Porto example. The absolute bias of an investigated estimator $\hat{Q}$ for the two-dimensional s.d. *Q* as a parameter is defined by $\|\hat{Q}{-Q\|}_{G}$. The empirical absolute bias and its standard error (SE) correspond to the mean and standard deviation of absolute biases in 100 replications, respectively. All simulations were carried out in Python using the PyDTMC library developed for analysing discrete time Markov chains (https://pypi.org/project/PyDTMC/). The codes and datasets of our simulation are available upon request.

Table 2 displays the simulation results for the small road network in Fig 1 using the Markov kernel of Fig 3 (We implemented this example in Python, see: https://github.com/rbesenczi/Crowd-sourced-Traffic-Simulator/blob/master/model-sources/Markovkernel/example_graph.py). The simulation parameters were *k* = 100, 200, 500, and 1000 number of trajectories with *n* = 3, 5, and 10 length. The absolute bias and its standard error do not depend on the length *n* and they are decreasing as *k* is increasing for both estimation methods. The latter is an expected result. Moreover, while for relatively small *k* the performance of the WLS and ML methods are similar, in the case of relatively large number of trajectories the ML estimator outperforms the WLS one a little bit. This phenomenon could be due to the asymptotic optimality of the ML estimator because the parameter *k* is enough large (1000) compared to the size of the road graph (5).

**Table 2:** Simulation results, absolute bias and SE (inside parenthesis), for the Markov kernel in Fig 3 on the road network in Fig 1. (*k*—number, *n*—length of trajectories).

k	n	ML	WLS
100	3	0.034 (0.0103)	0.034 (0.0109)
100	5	0.035 (0.0106)	0.034 (0.0103)
100	10	0.033 (0.0095)	0.033 (0.0094)
200	3	0.024 (0.0066)	0.026 (0.0066)
200	5	0.023 (0.0064)	0.024 (0.0067)
200	10	0.024 (0.0071)	0.025 (0.0070)
500	3	0.015 (0.0046)	0.017 (0.0049)
500	5	0.015 (0.0041)	0.017 (0.0049)
500	10	0.016 (0.0047)	0.017 (0.0049)
1000	3	0.010 (0.0032)	0.013 (0.0041)
1000	5	0.011 (0.0034)	0.015 (0.0044)
1000	10	0.010 (0.0030)	0.014 (0.0040)

In the second simulation scenario, a strongly connected subgraph, which contains 1000 vertices, of Porto’s road network was chosen (exported from the OSM, as well, GPS coordinates W8.6137, W8.5991, N411573, N41.1437). The entries of Markov kernel were generated randomly. The simulation parameters were *k* = 1000, 3000, and 5000 with *n* = 3, 5, and 10. In this scenario, the absolute bias and its standard error depend also only on *k* and are independent of the length *n*. However, there are significant differences between the performances of the two estimation methods (ML and WLS) related to the parameter *k*. On the one hand, the absolute bias of ML estimator is decreasing as *k* is increasing while it is constant for WLS estimator. On the other hand, the WLS estimator is better than the ML one in case of *k* = 1000 but worse in case of *k* = 5000. Since the former parameter setting is closer to the real traffic, this simulation corroborates the superiority of WLS method based on two-dimensional s.d. against the traditional maximum likelihood. Finally, in this scenario, the scale of the SE’s indicates that the WLS estimator is more stable than the ML one See Table 3.

**Table 3:** Simulation results, absolute bias and SE (inside parenthesis), for a part of Porto’s map with 1000 vertices. (*k*—number, *n*—length of trajectories).

k	n	ML	WLS
1000	3	0.166 (0.0559)	0.025 (0.0007)
1000	5	0.184 (0.1214)	0.025 (0.0007)
1000	10	0.169 (0.0938)	0.025 (0.0008)
3000	3	0.064 (0.1725)	0.023 (0.0005)
3000	5	0.070 (0.1665)	0.023 (0.0005)
3000	10	0.063 (0.1705)	0.023 (0.0005)
5000	3	0.016 (0.0150)	0.023 (0.0004)
5000	5	0.014 (0.0055)	0.023 (0.0004)
5000	10	0.014 (0.0126)	0.023 (0.0003)

We have also implemented the model in the OOCWC system in order to apply our simulation method for real large-scale problems. First, we have filtered the TTP dataset. Then, we have created the Markov kernel from the filtered dataset, so all nodes of the simulation graph will have the corresponding transition probability vector. We should note, however, that not all nodes can be found in the Markov kernel, because it can happen that the dataset does not completely cover the whole map, i.e., not all nodes are part of a trajectory. In this case, we set uniform distribution for the corresponding node. Finally, we had to modify the basic operation of the simulation algorithm. In the original implementation, the cars are moving on the map quite randomly. Now, a car selects the next node based on the transition probability vector of the current node. For this, we use the pseudo-random number generation engine from the Boost Random library that is based on the method presented in paper [74].

**Fig 9:**
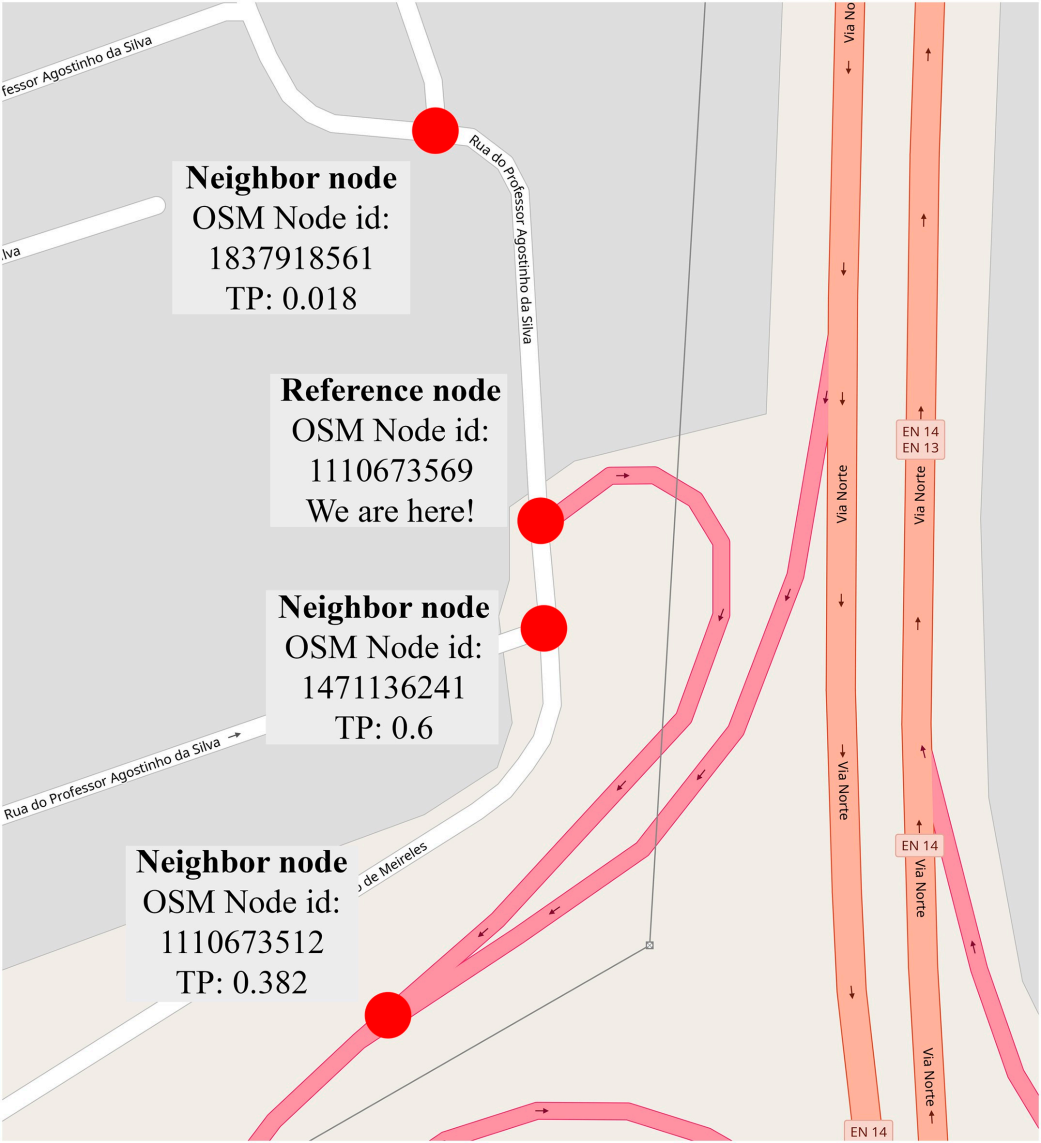
A visual explanation of transitions of intersection 1110673569. TP means transition probability, red dots indicate nodes. (Base map and data from OpenStreetMap and OpenStreetMap Foundation. Reprinted from OpenStreetMap under a CC BY license, with permission from OpenStreetMap, original copyright 2020. ^©^OpenStreetMap contributors. Annotated by the authors).

Let’s consider an example. We are at the graph vertex (or intersection) of OSM node ID 1110673569 (with GPS coordinates 41.1752185, -8.6231927). The total transitions of this node (i.e. the total trajectories that cross this intersection) in the dataset is 1,649. The transitions to the neighbor nodes are shown in Table 4. Please note that the actual transition probability (TP) is not the same as the ratio of the transitions to the neighbor node and the total transitions of the node which is called frequency (or ML) based transition probabilities. The actual transition probability comes from the Markov kernel of the whole graph. The two kinds of transition probabilities are also compared in Table 4 where the WLS based transition probabilities have been derived by our method. One can already see in this simple example that the difference between the two methods could be huge. This small example can be observed in Fig 9, as well.

**Table 4:** Transitions of intersection 1110673569. (TP—transition probability).

Neighbor node	# of transitions	ML based TP	WLS based TP
1471136241	1449	0.879	0.6
1110673512	170	0.103	0.382
1837918561	30	0.018	0.018
Sum	1649	1	1

**Fig 10:**
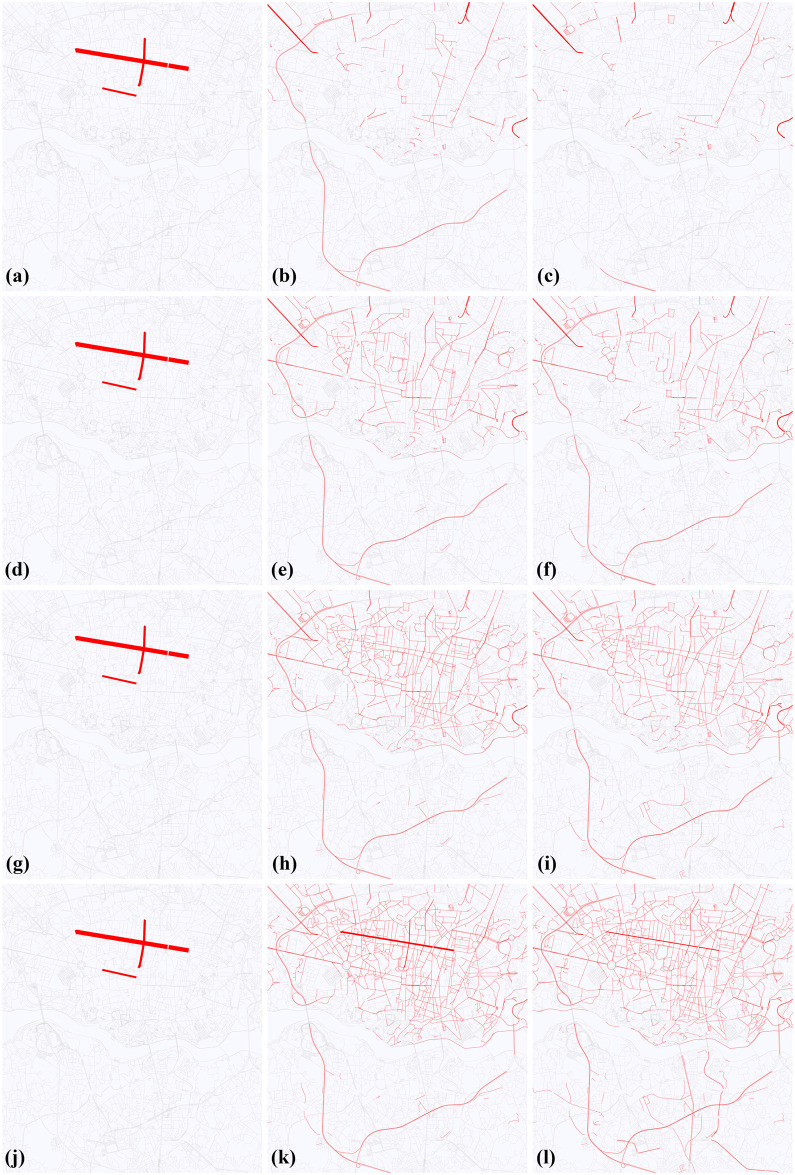
The change of the distribution of cars during the simulation (5,000, 10,000, 20,000 and 50,000 cars). The thickness of the street is proportionate with the number of cars on the street. a: Initial step (5,000 cars). b: After 30 mins (5,000 cars). c: After 60 mins (5,000 cars). d: Initial step (10,000 cars). e: After 30 mins (10,000 cars). f: After 60 mins (10,000 cars). g: Initial step (20,000 cars). h: After 30 mins (20,000 cars). i: After 60 mins (20,000 cars). j: Initial step (50,000 cars). k: After 30 mins (50,000 cars). l: After 60 mins (50,000 cars).

The initialization phase of the simulation adds traffic units to the map. Each unit is placed to an OSM node, i.e., on a vertex of the simulation graph. There exist two ways to do this, one is following a prescribed distribution (e.g. uniform), the other is following measured data. In our test case, we initialized simulations with fictional measured data. We put units only to the streets Rua de Antero de Quental, Rua da Constituição and Rua da Boavista (25.6%, 51.4%, 23% of the cars, respectively), i.e., the simulation starts from the traffic configuration which is concentrated on three nodes of the road graph. In addition, we can set the number of the simulation units. We run simulations with *k* = 5, 000, 10, 000, 20, 000, 30, 000 and 50, 000 units. The simulation starts when all simulation units are added to the map. Fig 10 shows the change of the distribution of cars during the simulation.

**Fig 11:**
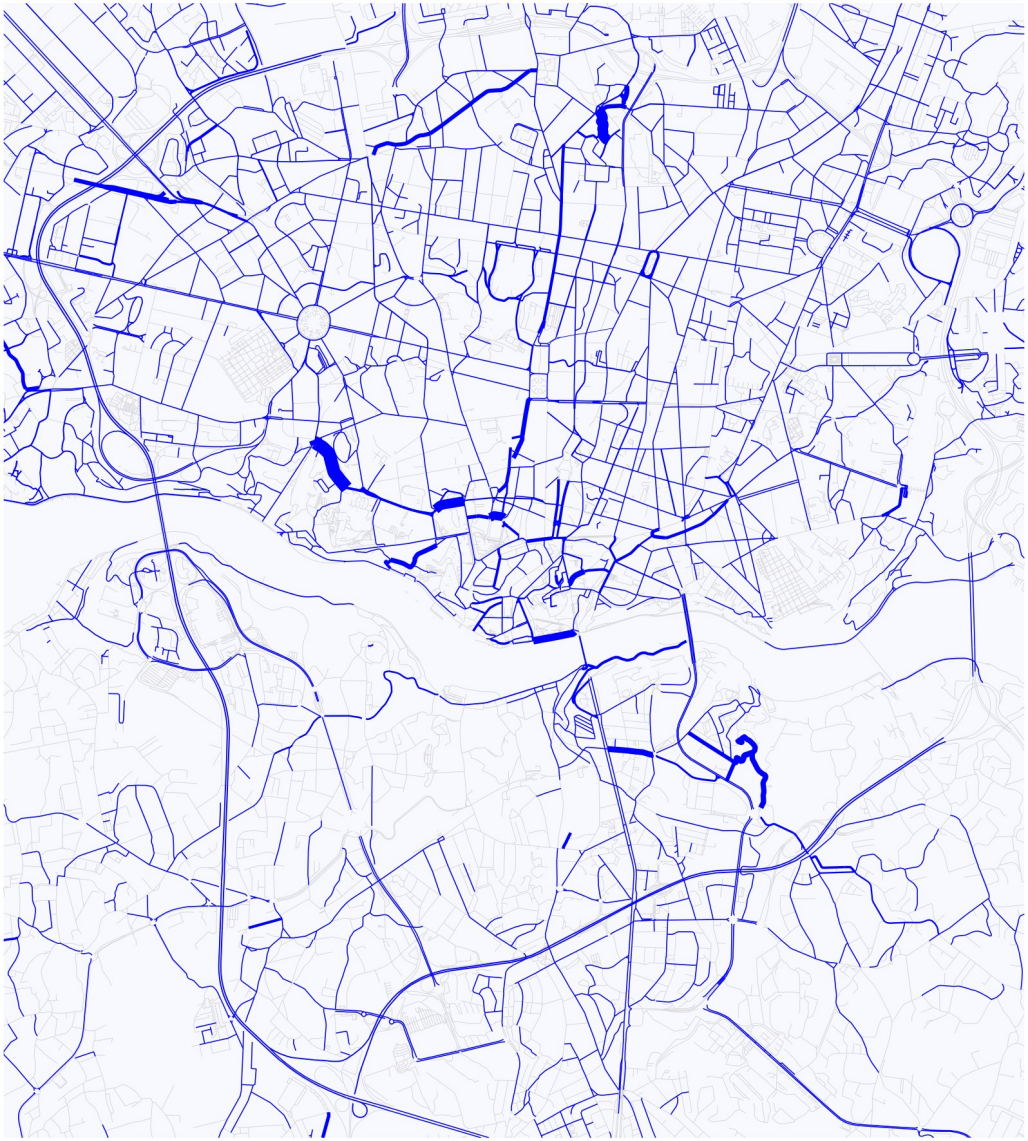
The stationary distribution of cars in Porto based on the TTP dataset.

The RCE produces a logfile that contains the position of every simulation unit in every simulation step. From this file, we calculate the number of cars by streets in every minute, so we can observe the change of distribution of the cars. In addition, we calculated the s.d. of cars for streets in the city of Porto, see Fig 11. This latter one tells us, what is the probability that a car is on a given street. It is worth noting the similarity between this figure and Fig 8a. The ticker line on Fig 11 corresponds to increasingly hot color on Fig 8a.

**Fig 12:**
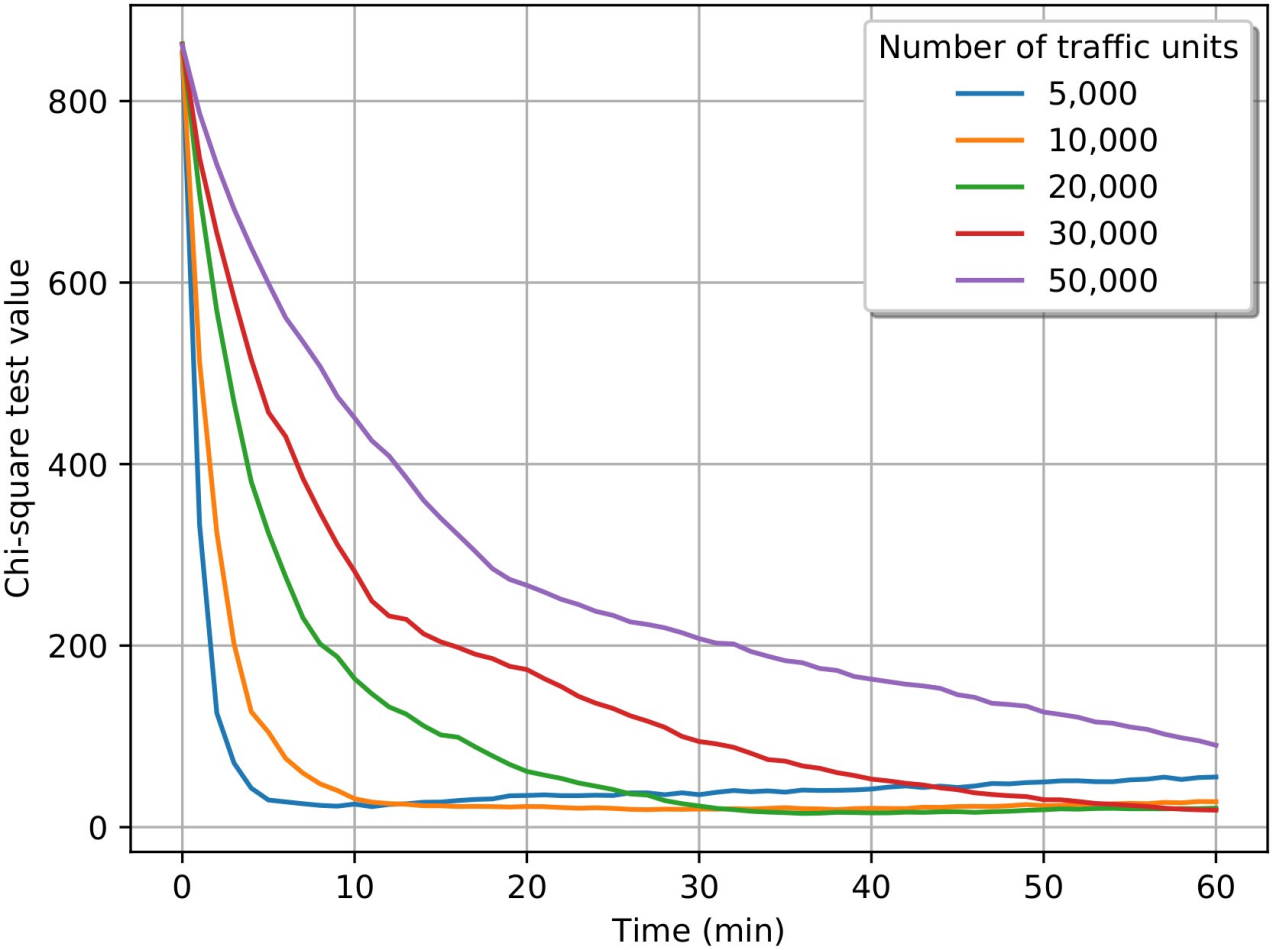
Chi-square test results.

To obtain a quantitative measure that describes the “goodness” of our simulation algorithm, we applied the Pearson’s chi-squared test. We expect, by the ergodicity of the Markov traffic, that during the simulation, independently from the initial distribution, within a certain time period, the distribution of the cars become close to the previously calculated stationary distribution. Fig 12 shows the test results. We can observe that in the first few minutes the test statistic is significantly high, meaning that the distribution of the cars is still far from the steady-state. However, after a time period that depends on the number of cars, the test statistic becomes low, meaning that the distribution became steady. One can observe that it takes more time to reach the steady-state with more traffic units, which is reasonable. Another notable trend is the case of 5,000 cars, where the line is elevating after reaching the steady-state. This can be caused by the low number of cars. The number of individual streets (named or unnamed, e.g. motorway junctions) is 2,194. 5,000 cars are simply not enough to reach and hold a steady-state in this type of simulation.

Finally, we should note some implementation details and possible drawbacks of this model that may have an impact on the model’s overall performance.

In small and medium graphs, the proposed algorithm and its implementation performs as it is expected. But in the case of our Porto example, where the graph has 33,961 nodes and 53,126 edges and the TP matrix is very sparse, numerical problems may occur. One problem can occur when we calculate the ${\hat{Q}}_{\text{WLS}}$ estimator and then TP matrix. For matrices with this size (34,000 x 34,000), we cannot solve the linear equation of the Lagrange vector in Theorem 2 always numerically, thus we could only use the least square solution for a numerically stable calculation. In some cases, this causes impossible numbers to present in the TP matrix, e.g. for a node, the TP vector is [1.17489, -0.174894], which is obviously impossible. It is interesting to note that the sum of these “malfunctional” TP vectors are 1 all the time, and mostly occurs if the node has a low number of transitions (less than 20). In such cases, we use the frequency based TP. Another numerical problem can occur when we calculate the s.d. ***π***, namely, negative values may present in the results. We need to handle this problem when we calculate the Pearson’s chi-squared test. We chose to shift every value of ***π*** until we get a sum of 1 for ***π***.

Some minor issues can occur with the map database and the differences between the Porto dataset and the OSM data. In some cases, we could not calculate a route between two consecutive trajectory points using OSM data. This can happen because of the imperfection of the OSM data or false GPS measurement. We handle this case by splitting the trajectory into pieces.

Another minor issue arises in the calculation of the Pearson’s chi-squared test. Since the OSM Porto map and the trajectory dataset do not cover each other perfectly, we only know the s.d. ***π*** for a subgraph of the whole map. During the simulation the units can traverse the whole map graph, so, it can happen that a traffic unit reaches an edge which is not part of the subgraph where we know the s.d. ***π***. During the calculation of the Pearson’s chi-squared test, we consider only those cars that are present on the road network, where the s.d. ***π*** is known.

## Conclusions

In this paper, we have described our traffic simulation model that is called “Markov traffic” based on tools from graph theory and Markov modeling. The aim was to provide a simulation method that is able to keep the distribution of the cars on the map in a steady-state on a large scale road network. We have proven that, under general assumptions, the stationary distribution (s.d.) is unique for any Markov transition mechanism on a wide class of road networks. An explicit formula has also been derived for the s.d. and the ergodicity of Markov traffic has also been proved.

We have shown that the s.d., with the related transition mechanism, can be explored from observed data based on sample trajectories. We have provided a statistical method and proved its optimality by simulation with which we can create the Markov kernel necessary to obtain a Markov traffic on a given road graph. Using this kernel, we can initiate traffic simulations that provide a s.d. of the cars on the map.

To provide an example for creating this kernel file, we have used a publicly available dataset, namely the Taxi Trajectory Prediction dataset. Our simulation uses OpenStreetMap, a free map database.

To test our theories, we have implemented the proposed model in our simulation program (RCE). We have run simulations and it has been proved to provide a s.d. on the map graph of Porto, Portugal. The whole project (including the RCE) is available for download (see https://github.com/rbesenczi/Crowd-sourced-Traffic-Simulator/blob/master/justine/install.txt). Some simulation video is available at the YouTube channel of the first author at http://bit.ly/2FRpPxL.

Future work will focus on the further validation of the assumptions of the Markov traffic model in cases of real traffic data and the possible applications of our simulation approach, e.g., modelling the pollution or energy consumption in a city due to multi-modal traffic with gasoline, diesel, electric and plug-in hybrid vehicles, as well as public transportation.

## Supporting information

S1 Appendix (PDF)
