# Supplementary material for: Large-scale simulation of traffic flow using Markov model
Source: PLoS One. 2021 Feb 9;16(2):e0246062. doi: 10.1371/journal.pone.0246062 (PMC7872230; doi:10.1371/journal.pone.0246062)
Supplement: S1 Appendix — (PDF) [file pone.0246062.s001.pdf]

S1 Appendix. Proof of Theorem 2. By the bias-variance decomposition, see Section 3.2 in [65], we have

$$\begin{aligned} \mathrm{SSE}(M, \boldsymbol{w} \mid \boldsymbol{N}) &= \sum_{i=1}^{k} w_i^{-1} \|N_i - w_i M\|_G^2 \\ &= \sum_{i=1}^{k} w_i^{-1} \|N_i - w_i N\|_G^2 + \|N - M\|_G^2. \end{aligned} \tag{15}$$

Clearly, if $\langle \cdot, \cdot \rangle_G$ denotes the inner product induced by the norm $\| \cdot \|_G$,

$$\begin{aligned} \|N_i - w_i M\|_G^2 =& \|N_i - w_i N + w_i N - w_i M\|_G^2 \\ =& \|N_i - w_i N\|_G^2 + \|w_i(N - M)\|_G^2 + 2\langle N_i - w_i N, w_i(N - M)\rangle_G. \end{aligned}$$

Thus, we have (15) since

$$\sum_{i=1}^{k} w_i^{-1} \|w_i(N - M)\|_G^2 = \sum_{i=1}^{k} w_i \|N - M\|_G^2 = \|N - M\|_G^2$$

and

$$\begin{aligned} \sum_{i=1}^{k} w_i^{-1} \langle N_i - w_i N, w_i(N - M)\rangle_G &= \sum_{i=1}^{k} \langle N_i - w_i N, N - M\rangle_G \\ &= \langle \sum_{i=1}^{k} N_i - \sum_{i=1}^{k} w_i N, N - M\rangle_G = 0 \end{aligned}$$

where we applied that $\sum_{i=1}^{k} N_i = N$ and $\sum_{i=1}^{k} w_i = 1$.

Minimizing SSE in its parameters M and $\boldsymbol{w}$ is equivalent by minimizing the right hand side of (15) partially: minimizing the first term with respect to $\boldsymbol{w}$; and minimizing the second term with respect to M. Since

$$\|N_i - w_i N\|_G^2 = \|N_i\|_G^2 + w_i^2 \|N\|_G^2 - 2 w_i \langle N_i, N\rangle_G$$

we have to minimize

$$\begin{aligned} \sum_{i=1}^{k} w_i^{-1} \|N_i - w_i N\|_G^2 &= \sum_{i=1}^{k} w_i^{-1} \|N_i\|_G^2 + \sum_{i=1}^{k} w_i \|N\|_G^2 - 2 \sum_{i=1}^{k} \langle N_i, N\rangle_G \\ &= \sum_{i=1}^{k} w_i^{-1} \|N_i\|_G^2 - \|N\|_G^2 \end{aligned}$$

under the constraint $\sum_{i=1}^{k} w_i = 1$. The Lagrange function is given as:

$$\ell = \sum_{i=1}^{k} w_i^{-1} \|N_i\|_G^2 - \|N\|_G^2 + \mu \left(\sum_{i=1}^{k} w_i - 1 \right)$$

where μ is the Lagrange multiplicator. For the first-order condition we have, for all $i = 1, \ldots, k$,

$$\frac{\partial \ell}{\partial w_i} = -w_i^{-2} \|N_i\|_G^2 + \mu = 0.$$

Thus, $w_i = \mu^{-1/2} \|N_i\|_G$, $i = 1, \ldots, k$, and hence $\mu^{1/2} = \sum_{i=1}^{k} \|N_i\|_G$ and $\widehat{w}_i = \|N_i\|_G / \sum_{j=1}^{k} \|N_j\|_G$, $i = 1, \ldots, k$, minimizes the first term of (15).

To minimize the second term in (15) define the estimator $\widehat{M} = (\widehat{m}_{uv})$ as a correction to the two-dimensional consecutive frequency matrix N in the following way: $\widehat{M} := N + R$ where $R := (r_{uv})_{u,v \in V}$ is a real matrix on V such that (i) $r_{uv} = 0$ if $(u,v) \notin E \cup S$ (i.e., $u \not\Rightarrow v$); (ii) $\widehat{m}_{v+} = \widehat{m}_{+v}$ for all $v \in V$; and (iii) $\|N - \widehat{M}\|_G^2 = \sum_{u \Rightarrow v} r_{uv}^2$ is minimal. Property (i) means that there is correction on the set $E \cup S$ only. Property (ii) means that $\widehat{M}$ is an unnormalized two-dimensional stationary distribution on G. Finally, property (iii) implies that $\widehat{M}$ is optimal in the least square sense. By Eq (10) one can see that (ii) implies

$$r_{v+} - r_{+v} - e_v + s_v = 0, \qquad v \in V. \tag{16}$$

Thus, the correction matrix R is given as a solution to the constrained optimization problem defined by

$$\frac{1}{2} \sum_{u,v:u \Rightarrow v} r_{uv}^2 \to \min$$

under the constraints (16).

The Lagrange function of this constrained optimization problem is given as:

$$\ell := \frac{1}{2} \sum_{u,v:u \Rightarrow v} r_{uv}^2 + \sum_{v \in V} \lambda_v (r_{v+} - r_{+v} - e_v + s_v)$$

where λ_v, $v \in V$, are the Lagrange multipliers. Taking the gradient of the Lagrange function we have the first-order condition for extrema as

$$\frac{\partial \ell}{\partial r_{uv}} = r_{uv} + \lambda_u - \lambda_v = 0, \quad \text{for all} \quad u,v \in V : u \Rightarrow v.$$

Thus, we have $r_{uv} = \lambda_v - \lambda_u$ for all $u \Rightarrow v$ which implies $r_{vv} = 0$ for all $v \in V$ and, for all $u, v \in V$,

$$\begin{aligned} r_{u+} &= \sum_{v:u \to v} (\lambda_v - \lambda_u) = \sum_{v:u \to v} \lambda_v - \deg^+(u)\lambda_u \\ &= \sum_{v \in V} a_{uv}\lambda_v - \deg^+(u)\lambda_u, \\ r_{+v} &= \sum_{u:u \to v} (\lambda_v - \lambda_u) = \deg^-(v)\lambda_v - \sum_{u:u \to v} \lambda_u \\ &= \deg^-(v)\lambda_v - \sum_{u \in V} a_{uv}\lambda_u. \end{aligned}$$

Thus, by (16), we obtain the linear equation for the vector $\boldsymbol{\lambda} \in \mathscr{F}$ defined by $\boldsymbol{\lambda} := (\lambda_v)_{v \in V}$:

$$\boldsymbol{s} - \boldsymbol{e} = (r_{+v} - r_{v+})_{v \in V} = (D - A - A^\top)\boldsymbol{\lambda} = L\boldsymbol{\lambda} \tag{17}$$

and the correction matrix given by $R = (\mathbf{1}\boldsymbol{\lambda}^\top - \boldsymbol{\lambda}\mathbf{1}^\top) \circ A$. Finally, it remains to prove that the Lagrange vector $\boldsymbol{\lambda}$ is uniquely defined as the solution to (17) under the constraint $\mathbf{1}^\top\boldsymbol{\lambda} = 0$. By Proposition 1 in [69], if G is strongly connected then the multiplicity of the eigenvalue 0 of L is 1 with eigenvector $\mathbf{1}$, and L is invertible on the $|V| - 1$-dimensional invariant subspace $\mathscr{S} := \{\boldsymbol{\alpha} \in \mathscr{F} \mid \mathbf{1}^\top\boldsymbol{\alpha} = 0\}$. By Eq (11), $\boldsymbol{s} - \boldsymbol{e} \in \mathscr{S}$ which implies that any solution to (17) is given by $\boldsymbol{\lambda} = L_{\mathscr{S}}^{-1}(\boldsymbol{s} - \boldsymbol{e}) + c\mathbf{1}$, where $c \in \mathbb{R}$, and $L_{\mathscr{S}}^{-1}$ denotes the inverse of L on subspace $\mathscr{L}$. Since $\mathbf{1}^\top\boldsymbol{\lambda} = c|V|$ we obtain that $c = 0$ and we finished the proof. □

Proof of formula 13. The formula (13) for the effective sample size follows from

$$n_{\mathit{eff}} := \mathbf{1}^\top\widehat{M}\mathbf{1} = \sum_{u,v:u \Rightarrow v} \widehat{m}_{uv} = \sum_{u,v:u \Rightarrow v} (n_{uv} + r_{uv}) = n - k + (\boldsymbol{d}^- - \boldsymbol{d}^+)^\top\boldsymbol{\lambda}$$

since

$$\sum_{u,v:u\Rightarrow v} r_{uv} = \sum_{u,v:u\to v} (\lambda_v - \lambda_u) = \sum_{v\in V} \deg^-(v)\lambda_v - \sum_{u\in V} \deg^+(u)\lambda_u = (\boldsymbol{d}^- - \boldsymbol{d}^+)^\top \boldsymbol{\lambda}.$$
